# An Analytical Framework on Utilizing Various Integrated Multi-Trophic Scenarios for Basil Production

**DOI:** 10.3390/plants12030540

**Published:** 2023-01-25

**Authors:** Ștefan-Mihai Petrea, Ira Adeline Simionov, Alina Antache, Aurelia Nica, Lăcrămioara Oprica, Anca Miron, Cristina Gabriela Zamfir, Mihaela Neculiță, Maricel Floricel Dima, Dragoș Sebastian Cristea

**Affiliations:** 1 Food Science, Food Engineering, Biotechnology and Aquaculture Department, Faculty of Food Science and Engineering, “Dunarea de Jos” University of Galati, Domnească Street, No. 111, 800008 Galaţi, Romania; 2 Faculty of Economics and Business Administration, “Dunarea de Jos” University of Galati, Nicolae Bălcescu Street, 59–61, 800001 Galati, Romania; 3 Department of Automatic Control and Electrical Engineering, “Dunărea de Jos” University of Galaţi, 47 Domnească Street, 800008 Galaţi, Romania; 4 Department of Biology, Faculty of Biology, Alexandru Ioan Cuza University, 700506 Iasi, Romania; 5 Department of Pharmacognosy, School of Pharmacy, Gr. T. Popa University of Medicine and Pharmacy, Universitatii Street Number 16, 700115 Iasi, Romania; 6 Institute for Research and Development in Aquatic Ecology, Fishing and Aquaculture, 54 Portului Street, 800211 Galati, Romania; 7 Faculty of Enginnering and Agronomy in Braila, “Dunarea de Jos” University of Galati, Domnească Street, No. 111, 800008 Galaţi, Romania

**Keywords:** aquaponics, basil, sturgeons, prediction models, forecasting models, growth bed

## Abstract

Here, we aim to improve the overall sustainability of aquaponic basil (*Ocimum basilicum* L.)-sturgeon (*Acipenser baerii*) integrated recirculating systems. We implement new AI methods for operational management together with innovative solutions for plant growth bed, consisting of *Rapana venosa* shells (R), considered wastes in the food processing industry. To this end, the ARIMA-supervised learning method was used to develop solutions for forecasting the growth of both fish and plant biomass, while multi-linear regression (MLR), generalized additive models (GAM), and XGBoost were used for developing black-box virtual sensors for water quality. The efficiency of the new R substrate was evaluated and compared to the consecrated light expended clay aggregate—LECA aquaponics substrate (H). Considering two different technological scenarios (A—high feed input, B—low feed input, respectively), nutrient reduction rates, plant biomass growth performance and additionally plant quality are analysed. The resulting prediction models reveal a good accuracy, with the best metrics for predicting N-NO_3_ concentration in technological water. Furthermore, PCA analysis reveals a high correlation between water dissolved oxygen and pH. The use of innovative R growth substrate assured better basil growth performance. Indeed, this was in terms of both average fresh weight per basil plant, with 22.59% more at AR compared to AH, 16.45% more at BR compared to BH, respectively, as well as for average leaf area (LA) with 8.36% more at AR compared to AH, 9.49% more at BR compared to BH. However, the use of R substrate revealed a lower N-NH_4_ and N-NO_3_ reduction rate in technological water, compared to H-based variants (19.58% at AR and 18.95% at BR, compared to 20.75% at AH and 26.53% at BH for N-NH_4_; 2.02% at AR and 4.1% at BR, compared to 3.16% at AH and 5.24% at BH for N-NO_3_). The concentration of Ca, K, Mg and NO_3_ in the basil leaf area registered the following relationship between the experimental variants: AR > AH > BR > BH. In the root area however, the NO_3_ were higher in H variants with low feed input. The total phenolic and flavonoid contents in basil roots and aerial parts and the antioxidant activity of the methanolic extracts of experimental variants revealed that the highest total phenolic and flavonoid contents were found in the BH variant (0.348% and 0.169%, respectively in the roots, 0.512% and 0.019%, respectively in the aerial parts), while the methanolic extract obtained from the roots of the same variant showed the most potent antioxidant activity (89.15%). The results revealed that an analytical framework based on supervised learning can be successfully employed in various technological scenarios to optimize operational management in an aquaponic basil (*Ocimum basilicum* L.)-sturgeon (*Acipenser baerii*) integrated recirculating systems. Also, the R substrate represents a suitable alternative for replacing conventional aquaponic grow beds. This is because it offers better plant growth performance and plant quality, together with a comparable nitrogen compound reduction rate. Future studies should investigate the long-term efficiency of innovative R aquaponic growth bed. Thus, focusing on the application of the developed prediction and forecasting models developed here, on a wider range of technological scenarios.

## 1. Introduction

### 1.1. The General Background of the Study

The European Green Deal strategy aims for the transformation of the European Union’s (EU) economy for a sustainable future. Specifically, targeting goals such as achieving zero pollution of water, restoring aquatic ecosystems and biodiversity as well as developing smart solutions for shifting to environmentally friendly food production systems. According to the European Commission (EC) [1], a sustainable blue economy offers many solutions to achieve the European Green Deal objective. Therefore, the EC [2] encourages the adoption of low-impact multi-trophic aquaculture systems and technologies, according to the belief that if managed in a sustainable way, aquaculture is a valuable, low-impact source of food and feed. However, this enforces the adaption of an increased sustainability approach within the future aquaculture sector development while maximizing the competitiveness and resilience of this sector. Integrated multi-trophic aquaculture, such as aquaponics, is considered, according to EC [2], a solution for achieving those goals, fact valid especially in the case of sturgeon’s farms for caviar production, since it increases their economic sustainability during the first years of activity as a result of second crop biomass production and commercialization.

Sturgeons are considered high-economic value fish species, suitable for rearing in recirculating aquaculture production systems (RAS). This is attributable to the possibility of real-time monitoring and control of both water quality parameters and biomass growth rate and behaviour, during their long-time production cycle. According to some authors [3] antecedent construction of barriers and dams on rivers has largely contributed to preventing the migration of sturgeons during the reproductive season. Amplified by the over-exploitation of the natural resources for caviar production, this significantly contributed to the decrease of wild stocks of sturgeons. As a result of this situation, according to other authors [4] sturgeon species are now listed in Annex I and II of the Convention on International Trade in Endangered Species (CITES) and they are protected from the over-exploitation in almost all range states. Therefore, rearing sturgeons within RAS will contribute to satisfying the market demand for sturgeon products. Additionally, it will aid the sustaining restocking programmes that, according to other authors [5] are of crucial importance for the stabilisation of wild sturgeon stocks. Most sturgeon aquaculture facilities target the production of caviar as a product, desideratum which requires, according to other authors [6], a long sturgeon breeding cycle (about 12–15 years), economic sustainability issues can occur since RAS have high operational costs, mostly due to high energy requirements [7]. Therefore, the integration of aquaponic modules into already existing RAS systems may increase the economic sustainability of sturgeon aquaculture by generating an extra income derived from the commercialization of plant biomass, as proven by other authors [8]. However, according to a previous study [9], to maximize the profit of multi-trophic aquaculture systems, the business strategy should include a careful analysis, aiming to limit both investment and operational costs associated with these engineering complex systems. Also, some authors [10] concluded that the lack of “know-how” and proper real-time water quality monitoring infrastructure could be a significant drawback for integrating aquaponics techniques into already existing RAS. According to other studies [11,12], the prediction of crop yield could improve significantly the production and commercialization strategy of aquaponic facilities, increasing their competitiveness and maximizing food supply at a suitable time and in the places where the market demand for food products rises.

### 1.2. Artificial Intelligence (AI) Based on Soft Sensors and Forecasting Models for Water Quality Monitoring, Fish and Plant Biomass Growth

Operational management in an industrial, large-scale, aquaponic system requires real-time monitoring of water quality parameters to maintain both the economic and ecological sustainability of the system and to produce high-quality crops. However, several authors [13] confirmed that, despite the latest developments in sensor technologies, real-time monitoring of water quality parameters is costly, increasing therefore both the investment and maintenance costs. Also, nowadays, the technical and technological development in water quality monitoring and control is mostly based on using complex network systems technologies that support the communication requirements necessary for control and automation between the complex hardware devices which are less efficient in terms of operational costs equipment and systems itself. Moreover, as revealed by other authors [14] standard water quality monitoring systems are complex, time-consuming and expensive since they use sophisticated equipment, and required special trained personnel for maintenance. In order to be appealing to stakeholders, the water quality monitoring and control platform must be limited to a low number of sensors, which must be easy to use, efficient to operate from an economic perspective, and affordable to be implemented considering the initial investment costs and reliable. Considering all these necessary demands, recent studies [13,15,16,17,18] have focused on the implementation of water quality platforms which integrates soft sensors that are able to generate virtual data, therefore assuring more cost-efficient monitoring and actuation of the process faster than the maximum sampling period available for the wireless sensor. The soft sensor development methods reveal a strong value in the choice of model and, as pointed out by previous studies [19,20,21]. Also, selecting the input sensors—predictors can be based on historical observations, as well as on the results obtained from applying selection algorithms such as principal component analysis (PCA) [22], Kernel partial least squares (PLS) or boosting-iterative predictor weighting PLS [23]. The reduction of multicollinearity is essential in order to improve the resulting prediction model accuracy. Therefore, it is essential to remove the variables which present the linear correlation between themselves to reduce the value of model coefficients and to make it more stable, assuring therefore a strong performance. According to some authors [21], early attempts of implementing prediction models implied both COD and BOD, targeting therefore to improve the operational management of the oxidation process within water treatment plants. The use of BOD as a dependent variable can be effective if a various range of parameters such as TSS [24], DO [25], TP [26], nitrogen compounds [25,27], pH [27] or COD [24] are used as independent variables. Biological filtration is considered a process which can highly benefit from the use of soft sensors since nitrogen compound sensors are very expensive and demand significant operational costs during the operational cycle. Other studies that target to predict dissolved oxygen (DO) concentration in water by using Artificial Neural Networks (ANN) and Adaptive-Neuro Fuzzy Inference System (ANFIS) as ML techniques and pH, BOD and water temperature as input variables [28]; ANN, ANFIS and Random Forest (RF) techniques and temperature, pH and conductivity (EC) as input variables [29]; ANN, ANFIS and Support Vector Machine (SVM) techniques and pH, BOD, COD, temperature, NH_3_ and flow rate as input variables [30]. The soft sensors for predicting nitrogen compounds can be a huge break-off in many industries, among which, aquaculture can be highlighted. The ammonia concentration in water was successfully predicted by using the following combinations: ANN technique and DO, BOD, COD, pH and suspended solids as predictors [31]; ANFIS technique and water temperature, EC, salinity, NO_3_, turbidity, K, Fe, Mg and PO_4_ concentration as predictors [32]; SVM with vary kernel function and BOD, DO and salinity as predictors [33], respectively. By analysing the already published papers existing in WoS database which used nitrogen compounds for developing soft sensors, it can be revealed that a number of 26 papers were published within 2015–2021, most of them focusing on NH_4_^+^, followed by NO_3_^−^ and TN. Recent studies [11,34,35,36,37] have focused on using machine learning (ML) to optimize aquaponics systems’ operational management. Thus, some authors [34] used an ML-based IoT system for optimizing nutrient supply in commercial aquaponic operations and identified NH_4_ and Ca as top nutrient predictors in am tilapia-lettuce coupled aquaponic system, reducing, therefore, the cost involved in regulating nutrient parameters with over 75%. Other researchers [36] presented an approach which involves the automatic control of the aquaponics system using an autoML algorithm, targeting to improve plant and fish growth and help monitor the system using a cloud platform. Recent study [36] concluded that in order to predict the optimal nutrients required for fish and plant growth in a single aquaponic set-up, Monte-Carlo (MC) techniques can be used for synthetic data generation, followed by power-transforming the numerical predictors and clipping the highest and lowest quantiles of data as feature engineering methods, while the Linear Support Vector Machine (SVM) with penalty parameter set to 1 was chosen as the ideal classifier. Prediction models based on ML, using ANN and ANFIS techniques, have been extended to target fish and plants [11] biomass as dependent variables [37], simplifying, therefore, the operational management within aquaponics systems. As a result, lettuce yield (fresh weight) prediction was developed [11] using four machine learning (ML) models, namely, support vector regressor (SVR), extreme gradient boosting (XGB), random forest (RF), and deep neural network (DNN). Prediction models for biomass growth in aquaponics systems can simplify the operational management but, in order to apply precocious management and to interfere, in time, in the production cycle, before the first obvious signs of production decline could appear, forecasting models could represent an efficient tool. Also, forecasting models for fish and plant growth can maximize the efficiency of the facility’s business plan, facilitating marketing, distribution, and supply operations. Several studies already demonstrated that forecasting models such as ARIMA, LSTM or Holt were successfully used in the fisheries and aquaculture sector [12,38,39,40,41]. However, no studies were performed for forecasting the growth of sturgeons reared in RAS. Developing forecasting frameworks based on already existing technologies could be feasible, especially if the production cycle is divided into small technological sequences.

### 1.3. Aquaponics Grow Media (GM)

The main types of aquaponic module setups, mentioned also by other authors [42,43], are deep water culture (DWC), nutrient film technique (NFT), and flood-and-drain (F&D) substrate systems. However, a recent study [12] revealed that the aquaponic substrate technique assures the highest rate of return, compared to DWC and NFT techniques. Considering that, according to various research studies [8,10], the cost of substrate represents a significant percentage of total investment costs performed to integrate aquaponics into already existing RAS, several attempts have been made by other authors [44,45,46] in order to identify a suitable material which should accomplish both economic and environmental sustainability desideratum. Thus, some authors [47] tested crushed stone number 3 (CS) and flexible polyurethane foam (FPF) as substrates to produce lettuce, integrated into a tilapia RAS and revealed that the use of CS assures a larger number of leaves, higher nutrients concentrations and increase production of lettuce biomass. Other media grow beds used in aquaponics, as revealed by some authors [48] are light-expanded clay aggregate (LECA), perlite or pumice, used both for root support and microbial substrate. Also, other authors [45] characterized substrate grow beds as sand and gravel as the most labor-intensive and highly exposed to clogging due to the deposition of detritus. Also, a recent research study [45] revealed that if increasing the production density of fish, most of the cultured crops tended to grow better if substrate aquaponics techniques is used, compared to DWC and NFT. Other GM which has been tested for aquaponic plant growth are volcanic stone, ceramic pellets, ceramic rings and nanorods [49] and, as a result, it had been concluded that nanorods GM experimental variant recorded the best results both in terms of plant growth and nutrients removal. Since environmental sustainability is an important desideratum in the European Green Deal and the circular economy is encouraged to be extended, recent research adopted unconventional substrates such as periwinkle shells and palm kernel shells [50] and concluded that superior plant growth and water nitrogen reduction were obtained, compared to the experimental variants where gravel substrate was used.

### 1.4. Aim and Hypothesis

The present research targets to elaborate an analytical framework, to improve the operational management and the sustainability of integrated multi-trophic systems for basil (*Ocimum basilicum* L.)-sturgeon (*Acipenser baerii*) production, in different technological scenarios, characterized by various nutrients inputs through the administrated fish feed. Therefore, AI-based solutions are used to develop soft sensors which will be able to predict the concentration of water quality parameters which are considered most important and cost-demanding at the same time. Also, forecasting models for both sturgeons and basil growth are elaborated to assist the operators and improve business planning and technological management. Targeting to encourage the use of agriculture by-products in the spirit of converting waste to wealth, the present study proposes an innovative substrate, which consists of *Rapana venosa* shells, to welcome the European Green Deal initiative for encouraging circular economy, as well as the increase of sustainability in all blue economy sectors.

**Figure 1:**
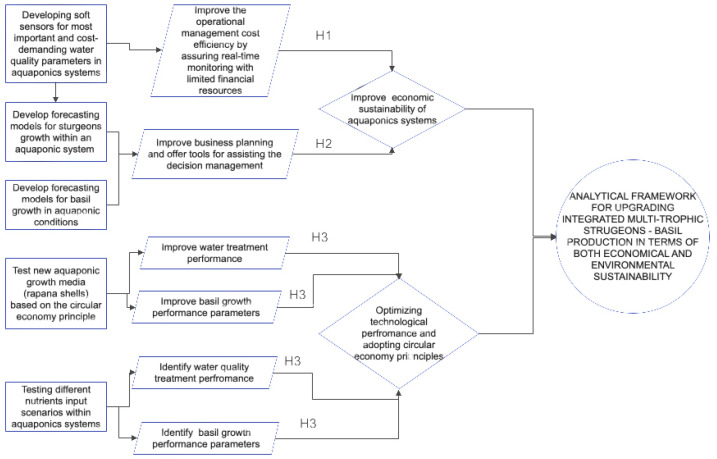
Research hypothesis interactions.

The following research hypothesis is designed, and their interactions are presented in Figure 1.

**H1.**  *Soft sensors could be successfully used in sturgeons-basil aquaponics multi-trophic systems in order to accurately predict the concentration of essential water quality parameters.*

**H2.**  *Forecasting models for fish and plant biomass could be used in order to identify the future dynamics of growth patterns in different technological scenarios.*

**H3.**  *Rapana venosa shells can represent a suitable substrate in order to compete with conventional GM, in sturgeons—basil aquaponics multi-trophic systems, considering different technological scenarios.*

## 2. Results and Discussion

### 2.1. Growth Performance of Both Acipenser baerii and Ocimum basilicum L. Biomasses

**Figure 2:**
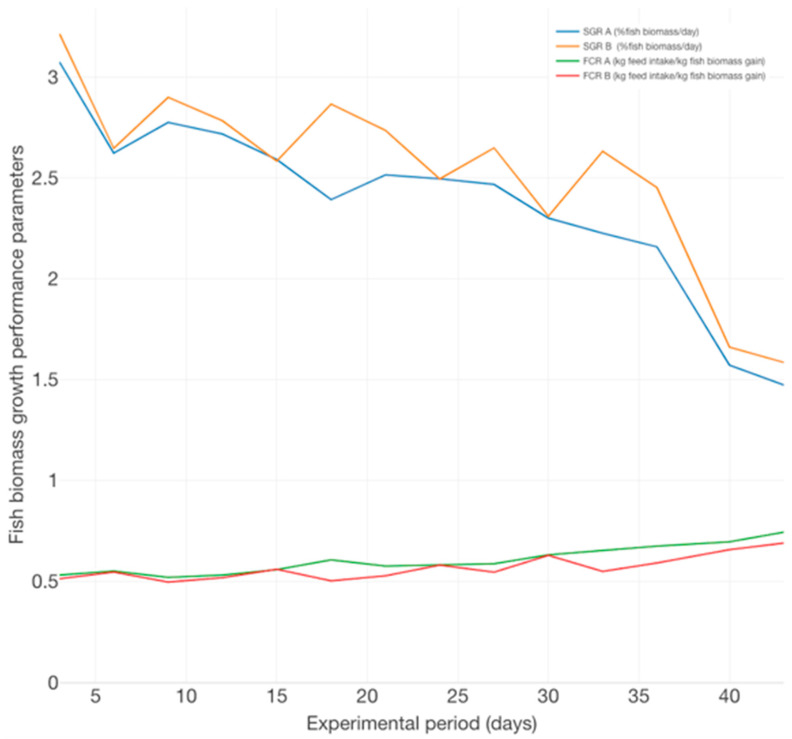
The trend of SGR and FCR during the experimental trial, for both A and B experimental; variants associated with different growth scenarios.

The average specific growth rate (SGR) expresses growth as the intuitively understandable per cent change in size per unit of time [51] and indicates superior fish productivity in the case of B variants (2.52%BW/day), compared to the A experimental variants (2.37%BW/day). The SGR dynamics indicate a decreasing trend, correlated with the increasing dynamics of food conversion ratio (FCR), a situation manifested especially during the last 5 days of the experimental trial (Figure 2).

From the perspective of feeding strategy efficiency, the FCR reveals better results for B (0.64 kg feed intake/ kg biomass gain) experimental variants, compared to A (0.68 kg feed intake/kg biomass gain), revealing the ability of fish organisms to utilize proteins, which positively affects growth rate [52]. Therefore, during the experimental trial, better cost efficiency is associated with the B variants technological scenario. However, this can be explained since fish from the A variants are in a superior development stage due to their higher average individual biomass (51 ± 5.3 g/ex), recorded at the beginning of the trial, compared to the B variants (30.93 ± 3.72 g/ex), a fact which confirms the results reported in other studies related to the decrease of both SGR and FCR as *Acipenser baerii* shifts to an advanced development stage during the production cycle.

**Figure 3:**
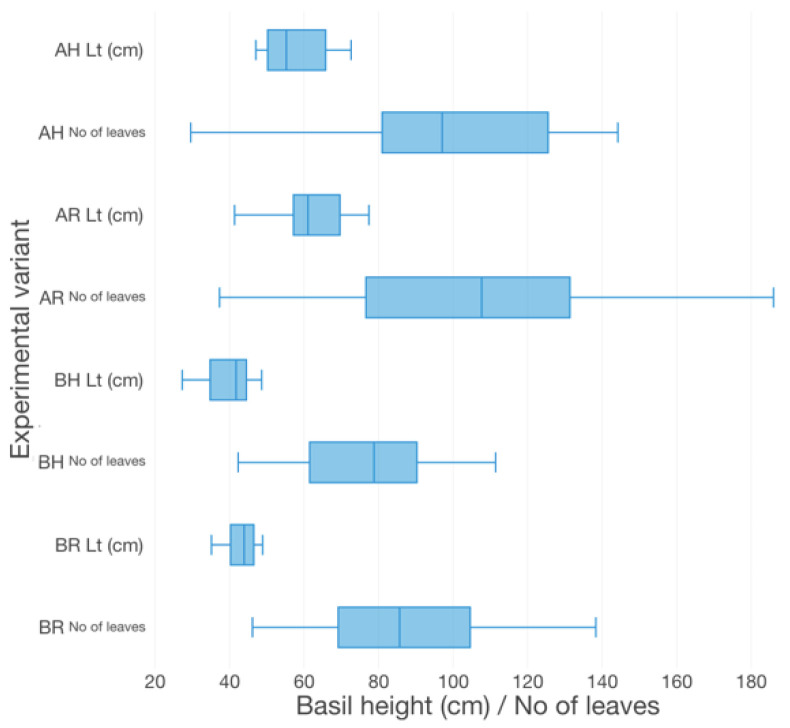
Boxplot for basil height and number of leaves for each experimental variant, at the end of the trial.

**Figure 4:**
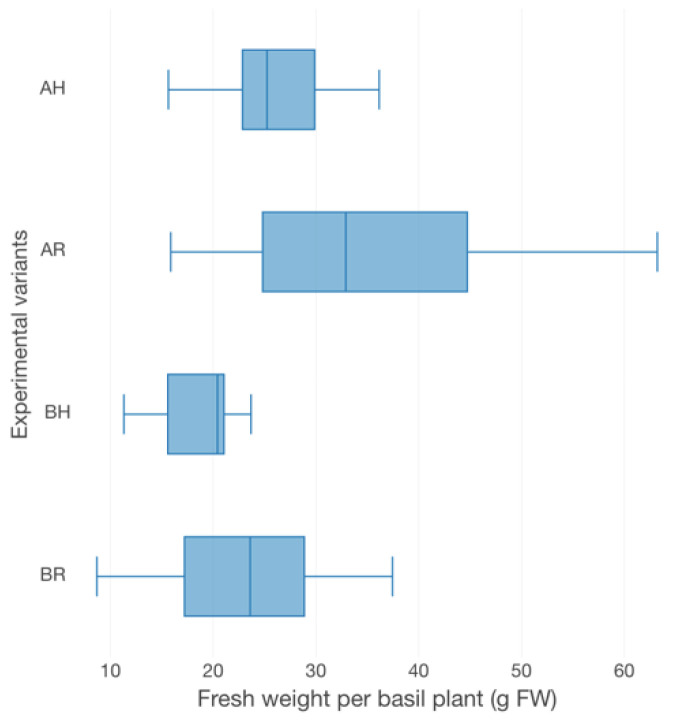
Boxplot for fresh weight per basil plant for each experimental variant, at the end of the trial.

**Figure 5:**
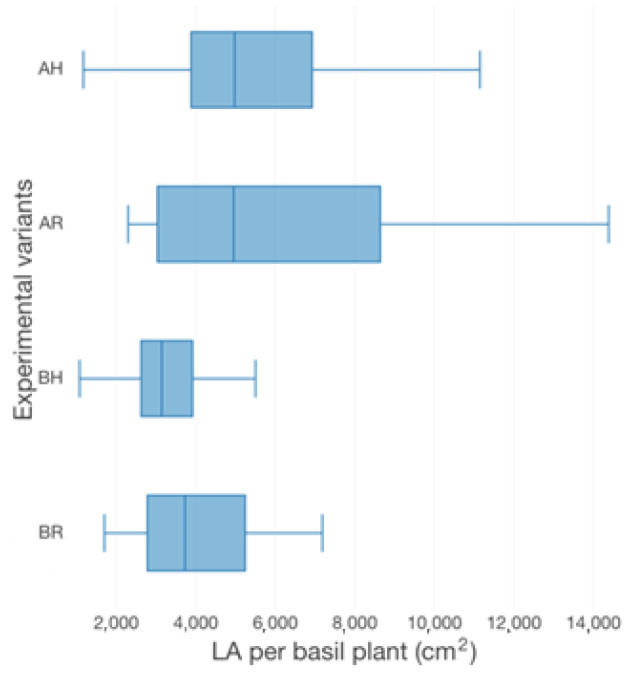
Boxplot for LA per basil plant for each experimental variant, at the end of the trial.

**Figure 6:**
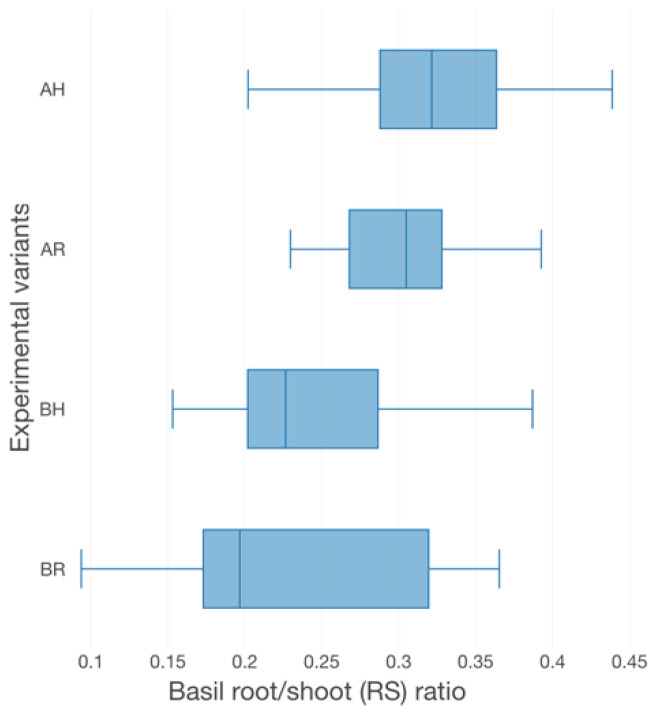
Boxplot for basil root/shoot ratio (R/S) for each experimental variant, at the end of the trial.

The basil growth performance was characterized by plant height (Figure 3), the number of leaves (Figure 3), fresh weight per plant (FW plant^−1^) (Figure 4), leaves area per plant (LA) (Figure 5) and root-shoot weight ratio (R/S) (Figure 6).

Therefore, in terms of basil height, the AR experimental variant registered the highest average value (57.67 ± 9.96 cm), followed by AH (61.40 ± 10.24 cm), BR (43.67 ± 5.59 cm) and BH (39.44 ± 6.42 cm). Significant statistical differences (*p* < 0.05) are registered between the variants which described different technological scenarios (AR, AH and BR, BH). However, no statistically significant differences (*p* > 0.05) are recorded between the GM treatments. The individual average weight reveals significant statistical differences (*p* < 0.05) between AR and BH (19.2 ± 4.71 g), BR (22.96 ± 8.05 g), respectively. However, the R GM assures better average fresh weight per basil plant (35.23 ± 14.43 g for AR and 27.27 ± 8.99 g for AH), compared to LECA (19.2 ± 4.71 g for BH and 22.96 ± 8.05 g for BR). No statistically significant differences (*p* > 0.05) were recorded between the experimental variants in terms of the number of leaves, LA and R/S. However, the high nutrients input generates from A variants, respectively the use of R GM in comparison to LECA, promotes better values of both leaves number per plant (17.31% higher in AR compared to BR, 23.26% higher in AH compared to BH, 2.88% higher in AR compared to AH and 5.81% higher in BR compared to BH) and LA (32.88% higher in AR compared to BR, 46.02% higher in AH compared to BH, 8.36% higher in AR compared to AH and 9.49% higher in BR compared to BH).

The results confirm the hypothesis related to which R GM can represent a comparable alternative to conventional GM as LECA, in aquaponics systems, since basil growth performance recorded superior values in AR compared to AH and BR, compared to BH, respectively. This finding is remarkable since, as mentioned in [53], different GM can affect the nutrient uptake of plants in aquaponics and, therefore, plants’ growth rate and plants’ quality. Some authors [54] revealed that water-caring nutrients can be better absorbed by plants if GM as LECA of coconut are used and, therefore, can promote vegetable biomass growth, compared to less water-caring GM as gravel, a fact which can be furthermore associated with R GM, used in the present study. However, since basil growth from R GM experimental variants was comparable with LECA GM variants, the water caring capacity of aquaponics GM cannot be considered a determinant factor in most cases. Thus, other authors [55,56] considered that microbial processes in the root zone as well as the substrate play a major role in assuring plant growth and nutrients fixation. According to some authors [57,58], in aquaponics systems, microbial activity mainly occurs on surfaces, and therefore, the majority of microbial communities are organized in biofilms. Thus, the shape, texture and type of GM exterior surface can be another characteristic that must be considered when characterizing as proper or not to be used in aquaponics, next to its caring water capacity.

Also, the findings confirm the results presented in previous studies [47,50] that highlighted the potential of using unconventional GM in aquaponics by revealing the superior growth performance of lettuce cultured on flexible polyurethane foam, compared with the biomass culture on conventional crushed stone GM [47], as well as the ability of by-products based GM as palm kernel shells and periwinkle shells to promote superior growth of pumpkin, compared to conventional gravel GM [50].

### 2.2. Forecasting Models for Both Acipenser baerii and Ocimum basilicum L. Biomasses Growth

#### 2.2.1. Forecasting Models for *Acipenser baerii* Biomasses Growth Based on ARIMA

Previous to the forecasting procedure, it is necessary to induce stationarity in the series. In order to stabilize the series variant, the square root transformation was used. The test ADF was used to investigate the stationary of time series, represented in Table 1.

**Figure A1:**
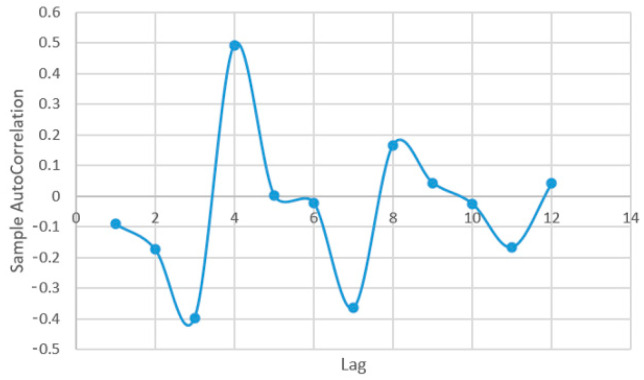
The AC with transformation in square roots—Series A.

**Figure A2:**
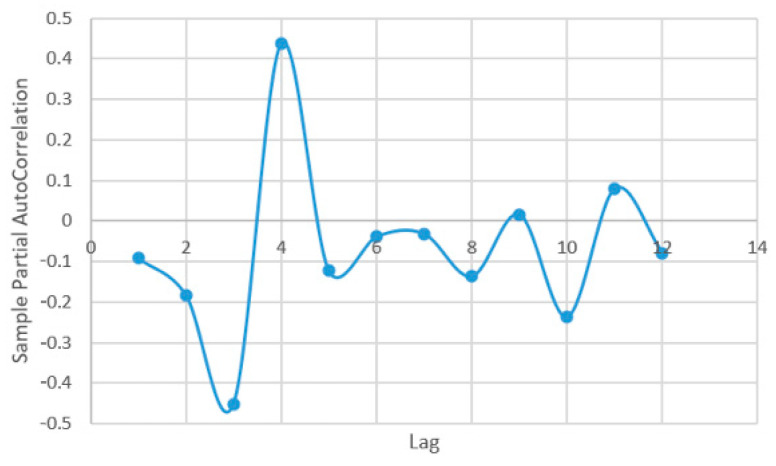
The PAC with transformation in square roots—Series A.

**Figure A3:**
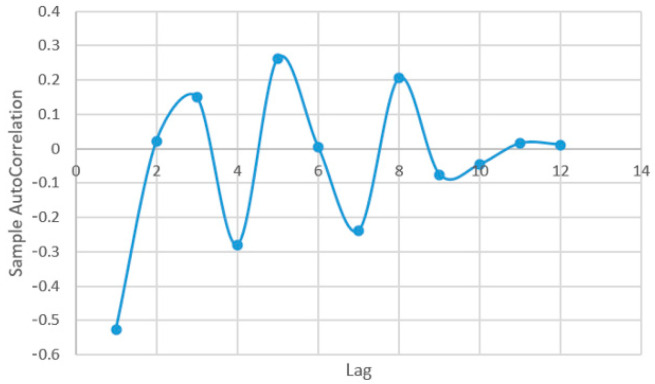
The AC with transformation in square roots—Series B.

**Figure A4:**
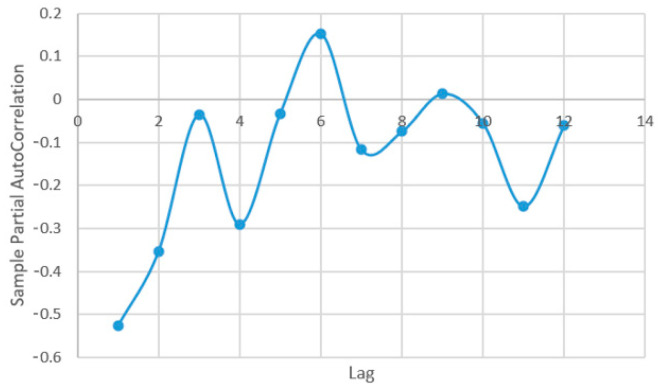
The PAC with transformation in square roots—Series B.

The second difference (d = 2) is optimum to be used in order to generate series stationarity. After executing this order 2 difference, the time series become stationary. After the series are stable, AC and PAC (Figure A1, Figure A2, Figure A3 and Figure A4; Appendix A) of 2nd order differences are used to estimate the parameters of the ARIMA model.

The orders of AC coefficients are useful to determine the mobile average (MA) order, while PAC order is used for establishing the autoregressive (AR) order. Orders selection for the autoregressive and mobile parts by using only correlogram visualisation can be inconclusive. This can be visualised by simulating different ARIMA models. Nevertheless, in order to select the best model, different orders of both ARIMA coefficients (p and q) can be used. In this case, several combinations with p = 1:2 and q = 1:3 were investigated, for the B model and the obtained results are represented in Table 2. As it is highlighted in Table 2, the models with the lowest Akaike coefficient are the ARIMA (2,2,2) for series A and the ARIMA (1,2,2) for series B.

After model identification and parameters estimation, the next step is to check if the residual values have a normal distribution. For the two aforementioned models, it was concluded that the residuals have a normal distribution. Thus, it can be stated that between the residual values no relation exists. The Jarque-Berra for the distribution analysis of residual series was applied and, therefore, it can be stated that both series of the residual variable are normally distributed, registering zero average and constant scattering (Jarque-Berra = 0.864 with *p*-value = 0.649 for series A and Jarque-Berra = 1.575 with *p*-value = 0.455 for series B).

**Figure 7:**
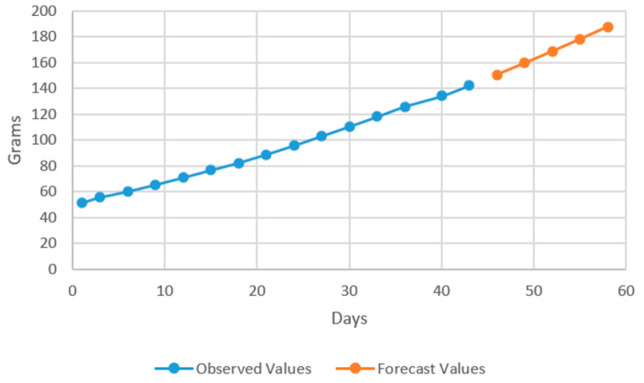
The evolution of *Acipenser baerii* biomass in A technological scenario.

**Figure 8:**
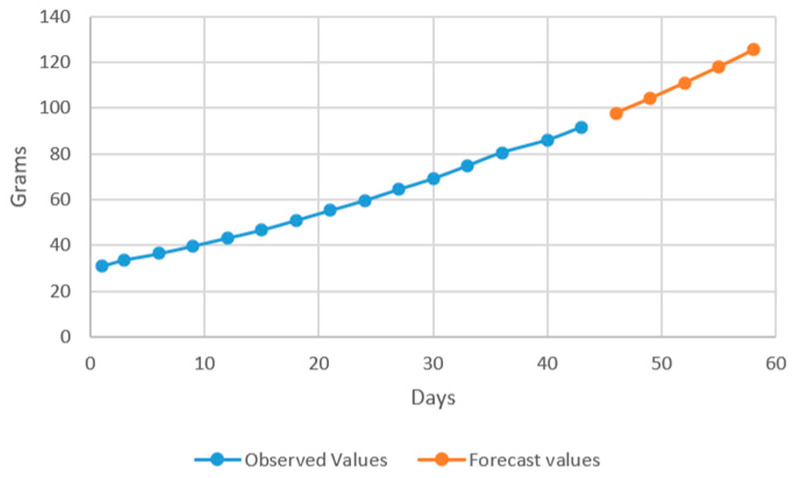
The evolution of *Acipenser baerii* biosmas in B technological scenario.

The aim of developing ARIMA models is to predict future values of the target variable considering the existing data. In this case, the model has two approaches: the values that are used for estimating the forecasting model and the forecasted values based on the identified model. In the models that resulted after performing the estimations (Figure 7 and Figure 8), the prediction was conducted over 5 periods (one period represents 3 days).

The models for *Acipenser baerii* biomass forecasting are presented in Equation (1), for A technological scenarios and Equation (2) for B scenario.

(1) $$d1a_{t}=0.274+0.116\;\times \;d1a_{t-1}-0.427\;\times \;d1a_{t-2}+1.193\;\times \;u_{t-1}-10.347\;\times \;u_{t-2}$$

(2) $$d1a_{t}=0.314-0.683\;\times \;d1a_{t-1}+0.024\;\times \;u_{t-1}+9.123\;\times \;u_{t-2}$$

where *d*1*a_t_* represents the 2nd order stationary series; *d*1*a_t_*_−1_, *d*1*a_t_*_−2_ the stationary series at a previous time; *u_t_*_−1_, *u_t_*_−2_ the mobile average order which is represented as predictions of the error variable.

The forecasting models have been successfully used by other authors [39,59], in order to predict fish production, confirming, therefore, ARIMA as the most recommended method in terms on forecasting accuracy. However, no studies that used ARIMA in order to predict sturgeons production performance were carried out until now. Therefore, the results can be considered a starting point for future research which will involve *Acipenser baerii* production forecasting in different technological scenarios.

#### 2.2.2. Forecasting Models for Both *Ocimum basilicum* L. Biomasses Growth Based on ARIMA

The time series data which implies basil height dynamics were stabilized by using square root transformation. Since it resulted that the time series was exponential, the logarithmation procedure was performed. To investigate the stationarity of the time series, the ADF test was performed, and the resulting data are presented in Table 3. As can be observed in Table 3, for the series A.H. and A.R. the series stabilizes at the 1st difference (d = 1), instead, the series B.H. stabilizes at the difference of the 3rd order (d = 3), and the B.R series stabilizes at the difference of the 2nd order (d = 2).

**Figure A5:**
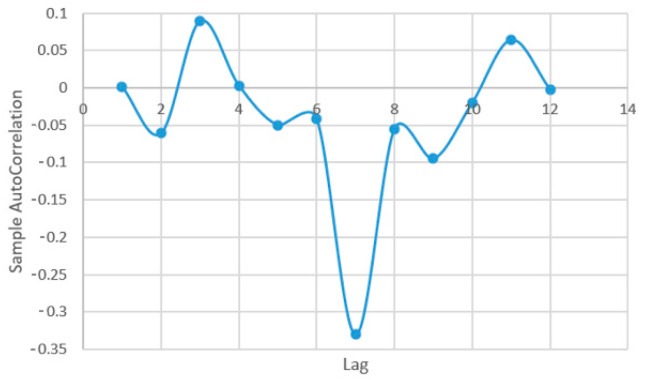
The AC with transformation in square roots—AH series.

**Figure A6:**
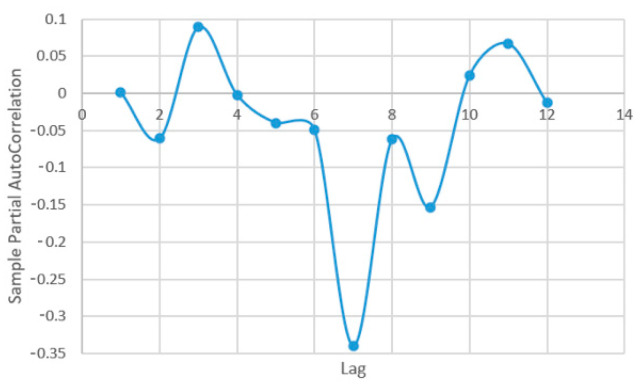
The PAC with transformation in square roots—AH series.

**Figure A7:**
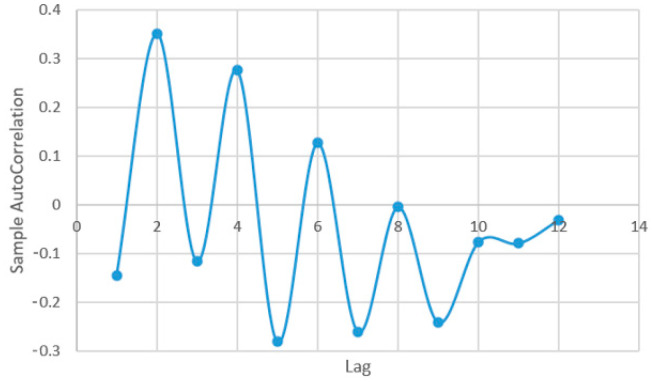
The AC with transformation in square roots—AR series.

**Figure A8:**
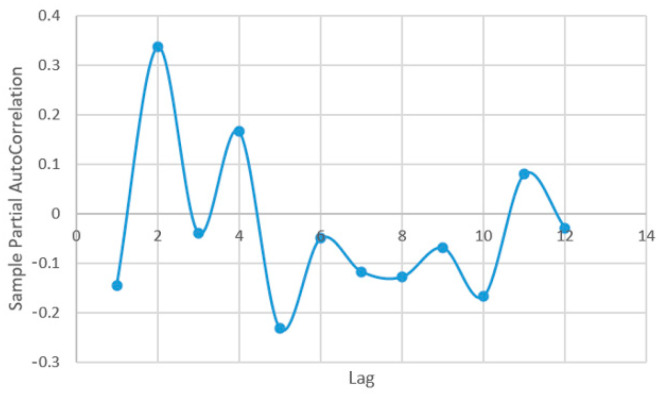
The PAC with transformation in square roots—AR series.

**Figure A9:**
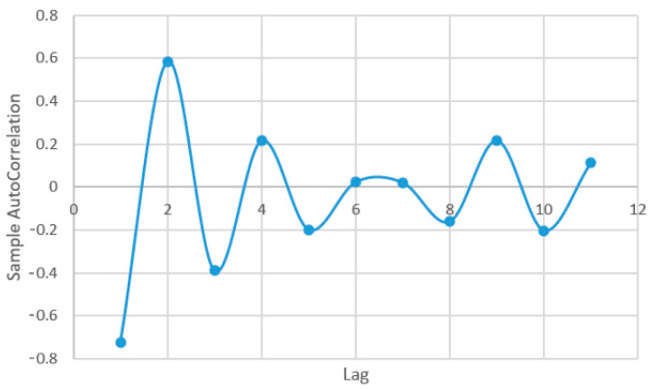
The AC with transformation in square roots—BH series.

**Figure A10:**
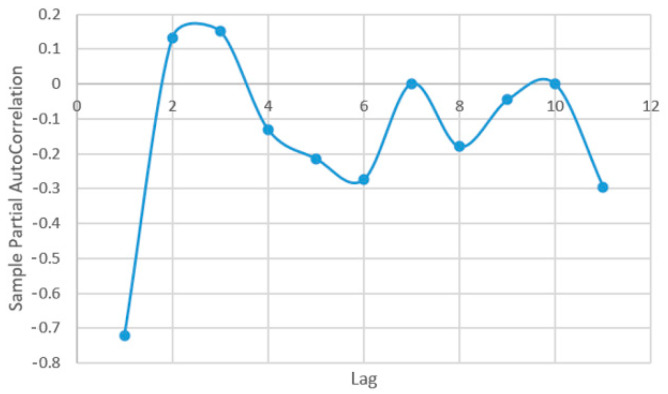
The PAC with transformation in square roots—BH series.

**Figure A11:**
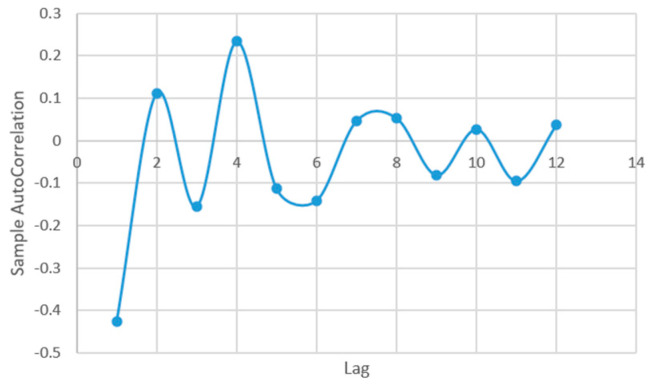
The AC with transformation in square roots—BR series.

**Figure A12:**
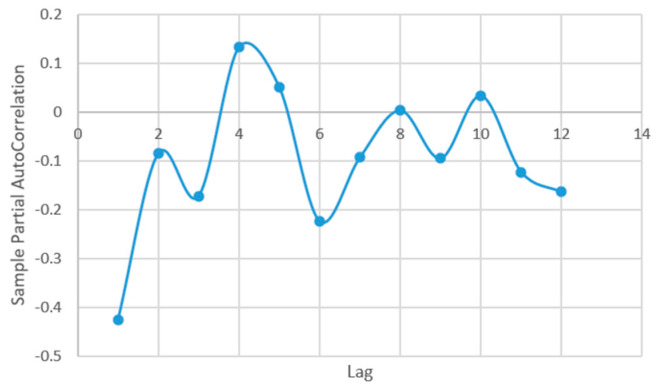
The PAC with transformation in square roots—BR series.

After different order differences have been performed, the time series becomes stationary. Since the series have been stabilized, the AC and PAC coefficients (Figure A5, Figure A6, Figure A7, Figure A8, Figure A9, Figure A10, Figure A11 and Figure A12; Appendix A) of the differences will be used to estimate the parameters of the ARIMA model.

The orders of the AC coefficients will be used for determining MA, while the PAC for determining AR order. Choosing orders for the AR and MA parts using only the correlogram view can sometimes be inconclusive. Thus, it is necessary to perform simulations for different ARIMA models. However, in order to select the most suitable model, different orders can be tested for both the ARIMA coefficients (p and q). Thus, the following combinations of models were performed: p = 1:2 and q = 1:2, in the case of the AH model; p = 1:2 and q = 1:2 for the AR model; p = 1:3 and q=1:3 for the model BH and p = 1:4 and q = 1:4, in the case of the BR model. The results obtained for the investigated models are presented in Table 4 together with the values of the Akaike coefficients.

As can be seen in Table 4, the models with the lowest Akaike coefficient are: ARIMA (1, 1, 2) for the AH series, ARIMA (2, 1, 2) for the AR series, ARIMA (2, 3, 2) for the series BH and ARIMA (2, 2, 2) for the series BR, respectively. The normal distribution of residuals is verified, and no relations are found between the residual values. The Jarque-Berra coefficient used in order to analyze the distribution of the residual series confirms the normal distribution of all series of residual values, having zero mean and constant dispersion (Jarque-Berra = 0.597 with *p*-value = 0.742 in the case of AH series, Jarque-Berra = 1.409 with *p*-value = 0.494 for AR series, Jarque-Berra = 0.558 with *p*-value = 0.756 for BH series and Jarque-Berra = 3.644 with *p*-value = 0.162 for BR series).

**Figure 9:**
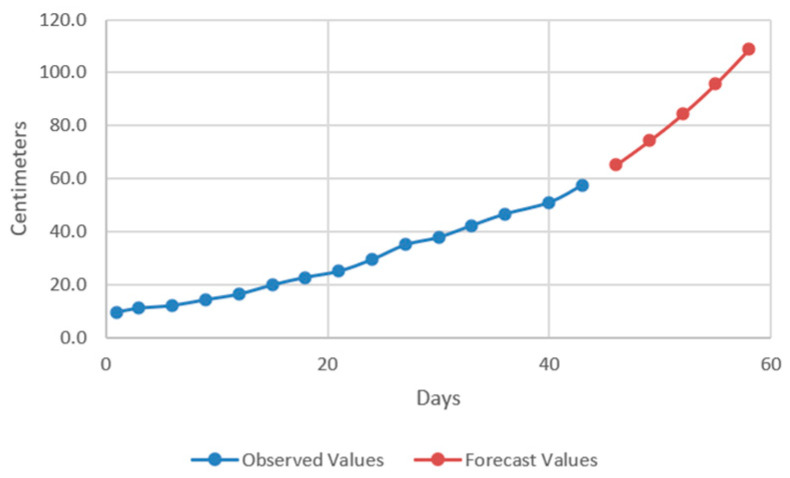
The evolution of basil shoot height in AH experimental variant.

**Figure 10:**
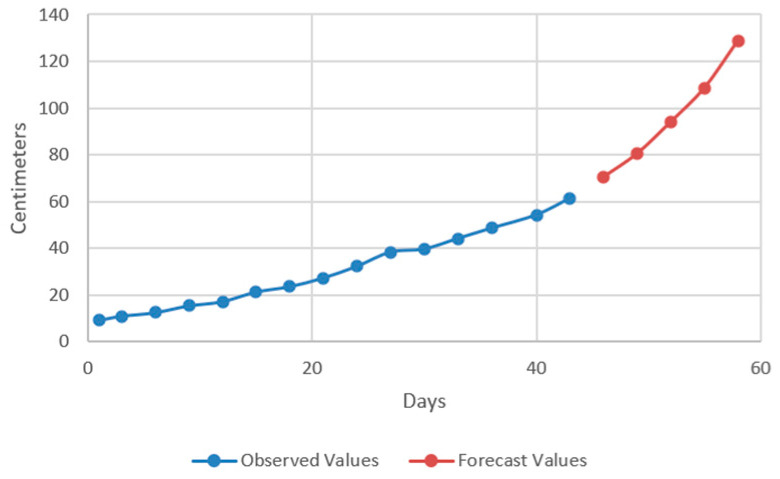
The evolution of basil shoot height in AR experimental variant.

**Figure 11:**
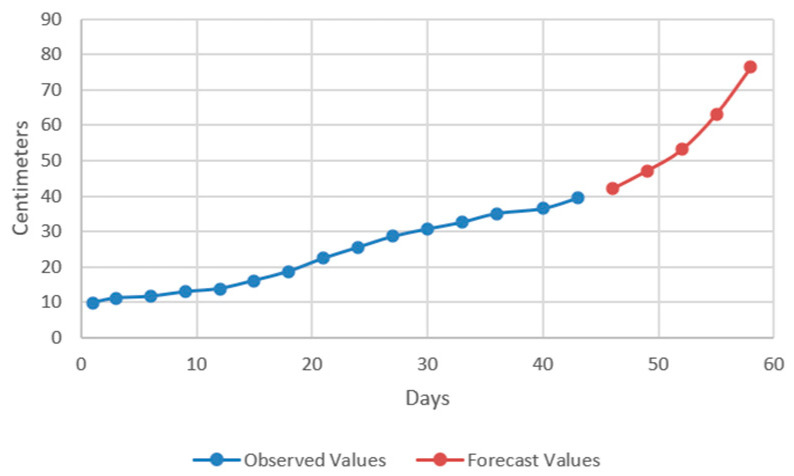
The evolution of basil shoot height in BH experimental variant.

**Figure 12:**
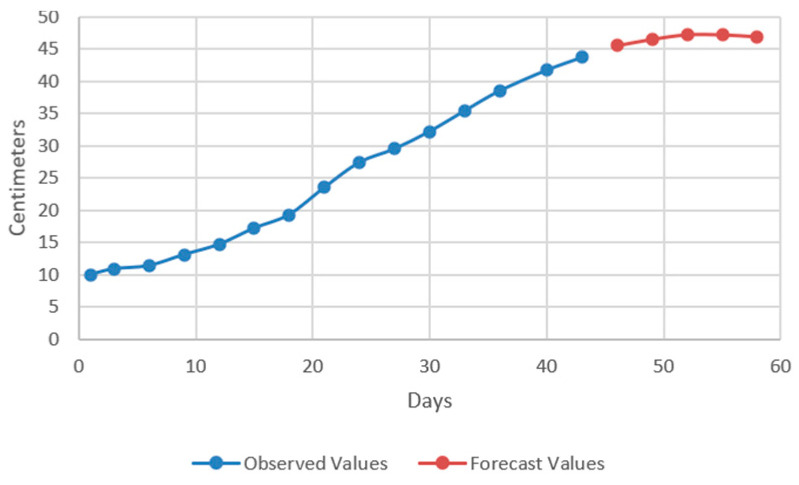
The evolution of basil shoot height in BR experimental variant.

In the models resulting from the estimates, the prediction was made for five periods (one period represents 3 days) (Figure 9, Figure 10, Figure 11 and Figure 12).

The models for basil shoot height forecasting are presented in Equation (3), for AH experimental variant, Equation (4) for AR, Equation (5) for BH and Equation (6) for BR.

(3) $$dlheight_{t}=0.055-0.572\;\times \;dlheight_{t-1}+0.97\;\times \;u_{t-1}$$

(4) $$dlheight_{t}=0.095-0.283\;\times \;dlheight_{t-1}+1.091\;\times \;dlheight_{t-2}+0.960\;\times \;u_{t-1}+5.771\;\times \;u_{t-2}$$

(5) $$dlheight2_{t}=0.001-0.180\;\times \;dlheight2_{t-1}+0.692\;\times \;dlheight2_{t-2}+3.145\;\times \;u_{t-1}+0.363\;\times \;u_{t-2}$$

(6) $$dlheight1_{t}=-0.003-0.005\;\times \;dlheight1_{t-1}+0.595\;\times \;dlheight1_{t-2}+1.41\;\times \;u_{t-1}+7.346\;\times \;u_{t-2}$$

where *dlheight_t_*—the stationary series at time t; *dlheight_t_*_−1_—the stationary series at time *t* − 1; *dlheight_t_*_−2_—the stationary series at time *t* − 2; *u_t_*_−1_, *u_t_*_−2_—residual variable regressions.

By applying the Equation (16) on the resulted forecasted data for basil shoot height, in order to forecast basil leaves area, 4 data series regressions have resulted, with high accuracy metrics (Equation (7) for AH experimental variant, Equation (8) for AR, Equation (9) for BH, Equation (10) for BR).

(7) $$\begin{array}{c} leaf_{t}= \end{array}\begin{array}{c} -609.9712\\ \left(0.0003\right) \end{array}\begin{array}{c} +123.2459\\ \left(0.0000\right) \end{array}\begin{array}{c} \;\times \;height_{t}\begin{array}{ccc} & & R^{2}=0.945 \end{array} \end{array}$$

(8) $$\begin{array}{c} leaf_{t}= \end{array}\begin{array}{c} -665.5813\\ \left(0.0014\right) \end{array}\begin{array}{c} + \end{array}\begin{array}{c} 126.1758\\ \left(0.0000\right) \end{array}\begin{array}{c} \;\times \;height_{t}\begin{array}{cc} & \begin{array}{cc} & R^{2}=0.916 \end{array} \end{array} \end{array}$$

(9) $$\begin{array}{c} leaf_{t}= \end{array}\begin{array}{c} -369.8742\\ \left(0.0060\right) \end{array}\begin{array}{c} + \end{array}\begin{array}{c} 138.1249\\ \left(0.0000\right) \end{array}\begin{array}{c} \;\times \;height_{t}\begin{array}{ccc} & & R^{2}=0.848 \end{array} \end{array}$$

(10) $$\begin{array}{c} leaf_{t}= \end{array}\begin{array}{c} -524.9811\\ \left(0.0035\right) \end{array}\begin{array}{c} +125.0911\\ \left(0.0000\right) \end{array}\begin{array}{c} \;\times \;height_{t}\begin{array}{ccc} & & R^{2}=0.891 \end{array} \end{array}\;$$

where *leaf_t_*—basil leaves surface at moment *t*; *height_t_*—basil height at moment *t*.

**Figure 13:**
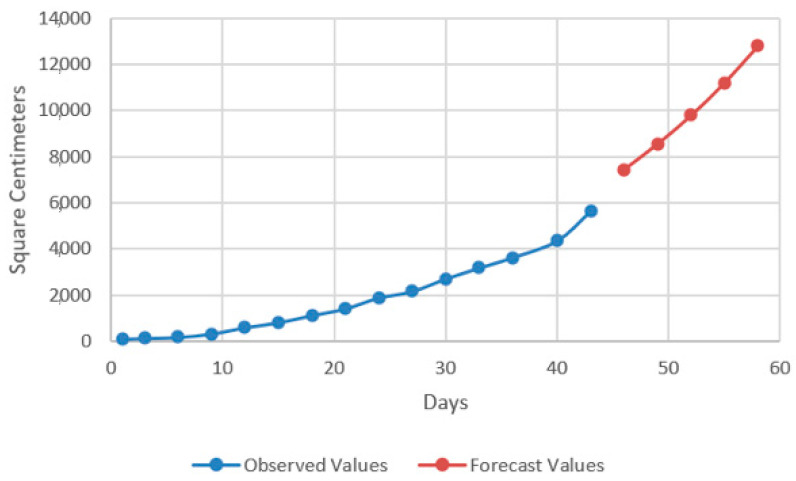
The evolution of basil leaves area in AH experimental variant.

**Figure 14:**
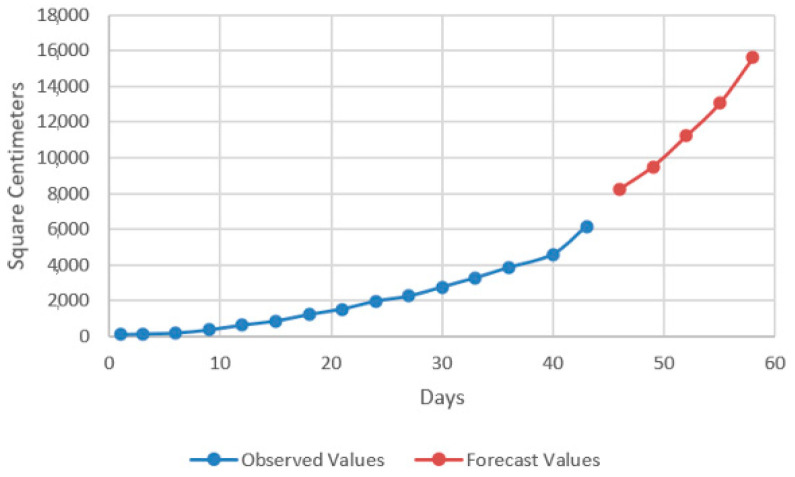
The evolution of basil leaves area in AR experimental variant.

**Figure 15:**
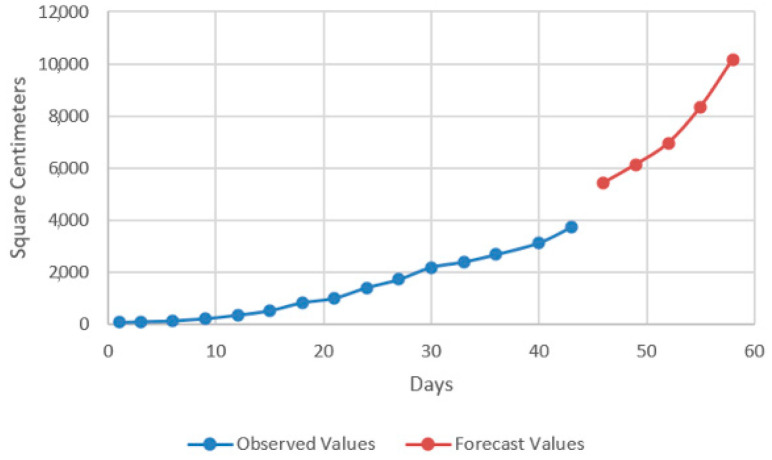
The evolution of basil leaves area in BH experimental variant.

**Figure 16:**
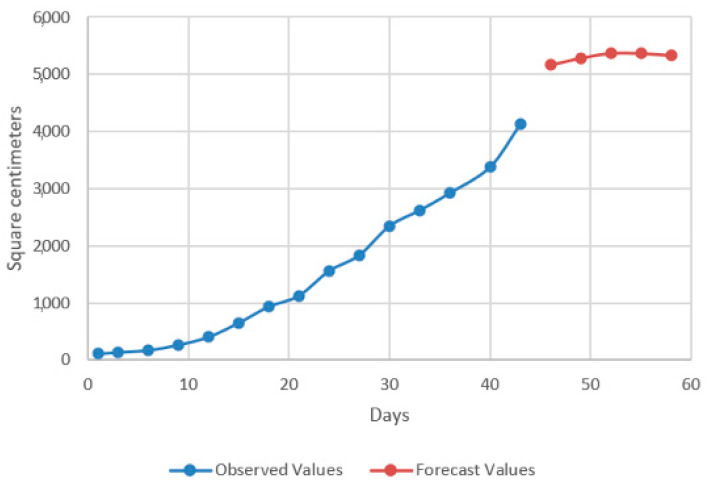
The evolution of basil leaves area in BR experimental variant.

Thus, by analysing the Equation (3) it can be stated that each 1 cm increase in basil height will lead to an increase of 123.25 cm^2^ in leaves area at AH, 126.18 cm^2^ in leaves area at AR, 138.13 cm^2^ in leaves area at BH and 125.09 cm^2^ in leaves area at BR. The forecasted dynamics, based on finding resulted by applying the Equations (3)–(6) are presented in Figure 13, Figure 14, Figure 15 and Figure 16.

Thus, the forecasting models reveal a significant increase trend on basil leaves area in the next 10 days, fact valid for AH, AR and BH, respectively (Figure 13, Figure 14 and Figure 15). However, the forecasting values for AR leaves area indicates a constant dynamic in the last 5 days of the forecasted period (Figure 16). Previous studies [60] have indicated ARIMA, ARIMAX and exponential smoothing as proper methods for plant growth forecasting. However, the results revealed that both ARIMA and exponential smoothing recorded higher RMSE, compared to ARIMAX, most probably due to the lack of regressors effect within prediction.

### 2.3. Water Quality and Nitrogen Compounds Reduction Capacity

Water quality parameters, monitored both at the inlet and outlet of aquaponics modules, were within the recommended range for *A. baerii* growth, as stated by other authors [61]. Thus, it can be observed (Table 5) that in the case of A technological scenario, the R GM experimental variant recorded higher concentrations in technological water for all nitrogen compounds (N-NH_4_, N-NO_2_, N-NO_3_), as well as for P-PO_4_, Ca, Mg, K and EC (Table 5). However, the concentrations of Fe and TOC, as well as the value of pH were lower in AR, compared to AH experimental variant (Table 5). Thus, it can be stated that the conventional GM performs better in terms of water treatment capacity and provides better conditions for aquaponic growth basil nutrient absorption. Also, the lower pH value and DO concentration recorded at H GM, corroborated with the higher Redox potential, TOC and COD concentrations (Table 5), indicates a superior accumulation rate of organic matter at the level of conventional light-expanded clay aggregated GM, compared to the R GM. Thus, even if H GM offers better performance in terms of nutrient retention, this can decrease based on long-term usage due to consecutive production cycles. Thus, the advantages of R GM could be revealed in time, especially in terms of reducing the operational costs for GM maintenance. 

**Figure 17:**
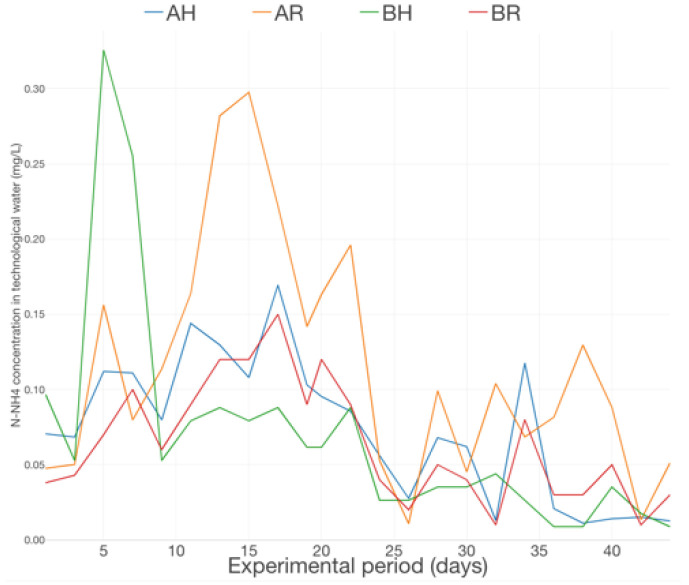
The dynamics of N-NH_4_ concentration in technological water for each of the experimental variants.

**Figure 18:**
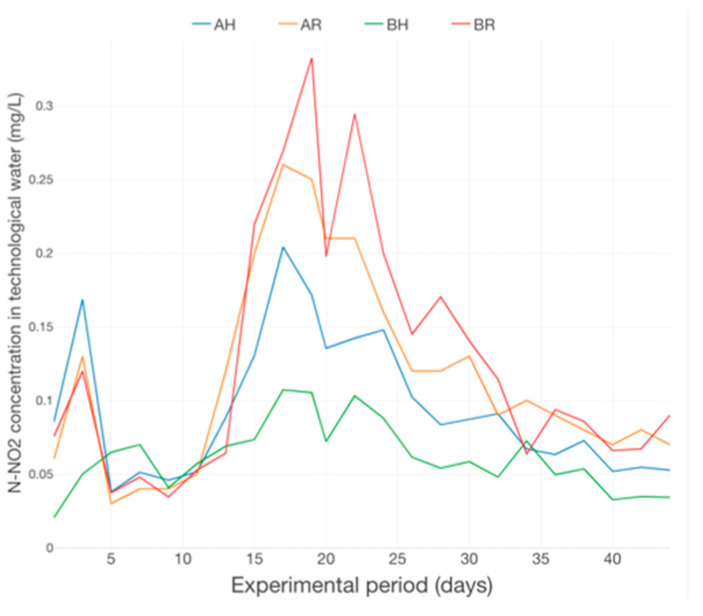
The dynamics of N-NO_2_ concentration in technological water for each of the experimental variants.

The dynamics of N-NH_4_ in technological water reveal an increasing trend in the 1st part of the production cycle (first 2 weeks), emphasizing 2 maximum peaks, after 5 days and, moreover, after 14 days from the beginning of the experimental trial (Figure 17). In the case of N-NO_2_ concentration dynamics (Figure 18), the maximum peaks were recorded in the middle of the experimental period, followed by a decreasing trend until near the end of the experimental trial and revealing a relatively constant evolution in the last 5 days of the basil production cycle.

**Figure 19:**
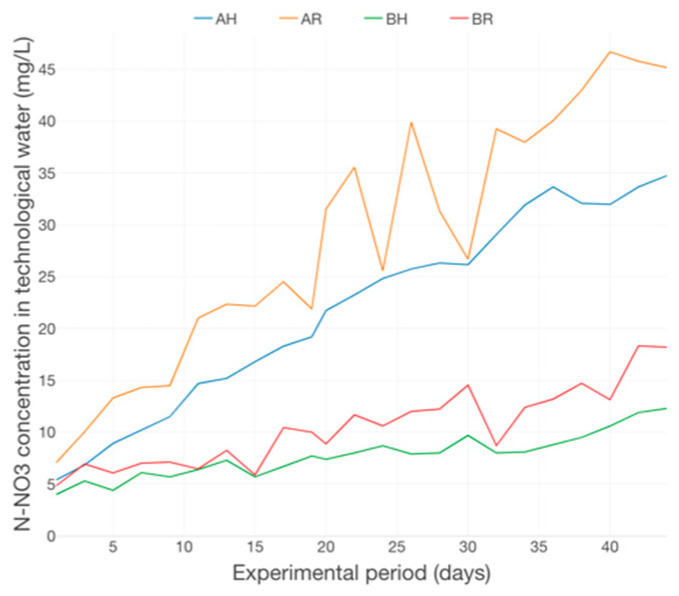
The dynamics of N-NO_3_ concentration in technological water for each of the experimental variants.

**Figure 20:**
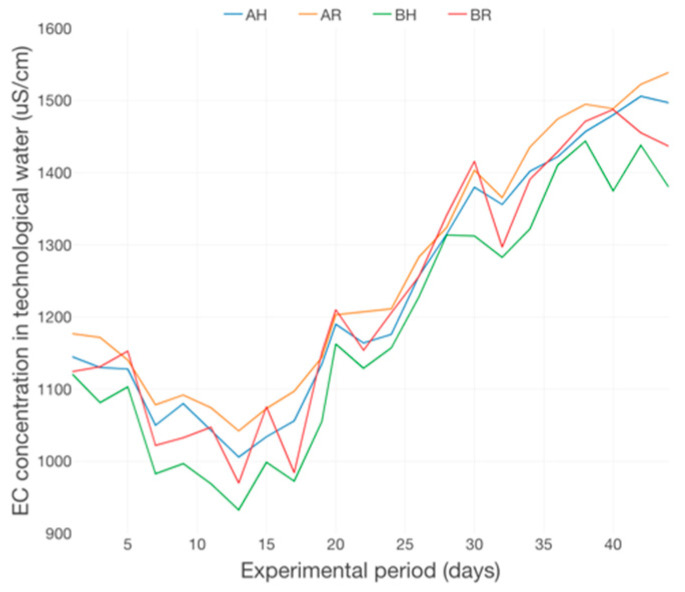
The dynamics of EC concentration in technological water for each of the experimental variants.

The N-NO_3_ dynamics emphasize a significant upward trend, revealing high differences between the variants which are part of different technological scenarios, A and B, respectively (Figure 19), a situation that highlights the accumulation tendency of this nitrogen compound and its positive relation with the fish feed inputs and the degree of intensivity associated to the fish rearing technology applied within an aquaponics system. This finding is confirmed by the EC highly increasing trend, manifested especially in the 2nd part of the experimental period (Figure 20).

The increasing tendency of EC during the experiment was also reported in other studies for example from 620 to 840 µS/cm in an aquaponic system (fish—tilapia; plants—basil) [62]. It is considered that the increase of EC during the experimental period is due, mainly, to the daily nutrient come from the fish feed.

**Figure 21:**
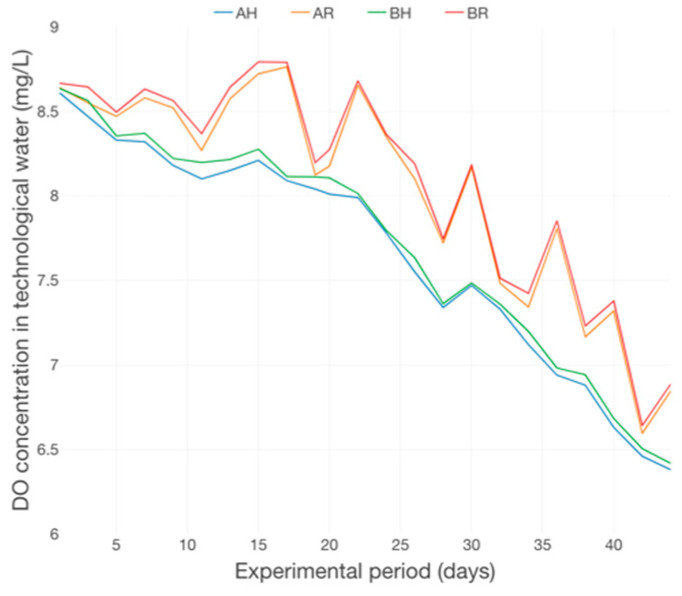
The dynamics of DO concentration in technological water for each of the experimental variants.

**Figure 22:**
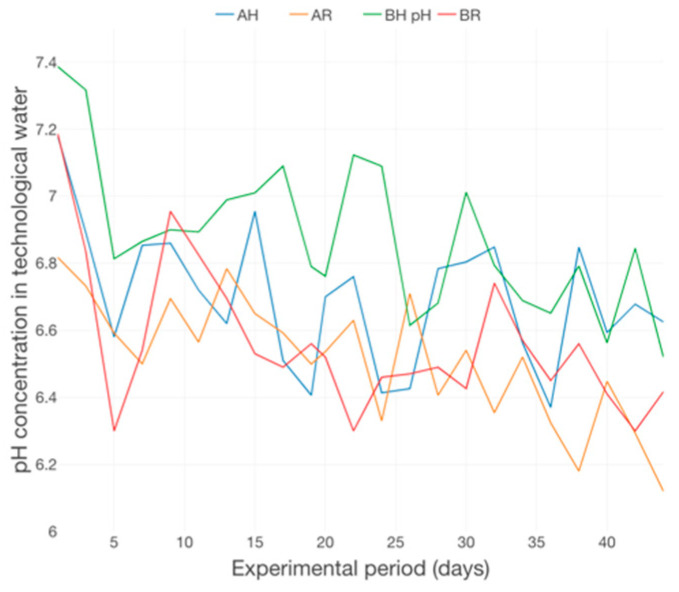
The dynamics of pH in technological water for each of the experimental variants.

The decreasing trend of DO concentration (Figure 21), correlated with a small decreasing trend of pH (Figure 22) could indicate an accumulation of organic matter, during the experimental period, at the level of GM, a fact more related to the type of GM used, rather than with the fish feed input. This can be related to the free surrounding space (FSS) presented around a unit of LECA, which is definitely lower compared to the FSS of a rapana shell, part of R GM, a fact that attributes a better mechanical filtration performance to H GM and, therefore, the vulnerability of being associated to a higher degree of organic matter accumulation during long-periods of consecutive production cycles.

Water quality is a primary consideration for aquaponic crop production, especially in a recirculating aquaponic system. Deterioration of water quality parameters not only affects fish physiology, growth rate, and feed efficiency [63], but also affects plant crop performance, quality and/or yield, and N use efficiency. Yang and Kim [64], reported that regardless of management regimes, DO in aquaponics averaged at 7 mg/L, which was slightly above the tolerance limits of 6 mg/L [65] and with 30% higher than 5 mg/L, which is suggested for aquaculture in terms of DO level [66].

Given that the nitrifying bacteria have an optimal range of DO (4–8 mg/L) to promote nitrification process [67], the DO levels from our experiment (7.53–8.09 mg/L) were sufficient in aquaponic system.

Also, was reported that the accurate pH ranges are 6–9 for fish growth, 5.5–6 for plants and 7–8 for nitrifying bacteria [68], so we can say that pH between 6.51 ± 0.19 and 6.88 ± 0.22 is considered an ideal compromise for aquaponics system. In case of Alacorn [69], the values of DO and pH fluctuated from 5.0 to 9.0 mg/L, respectively 6.2–8.2 upH.

Therefore, the pH changes recorded in our study were considered mainly due to the differences in water chemistry affected by the treatment, but the values are included in the optimal range for the growth of plants and fish (Table 5, Figure 19 and Figure 20). The tendency of lower values of pH may be partly due to a higher release of carbon dioxide from increased respiration of fish in the system derived from more active growth.

**Figure 23:**
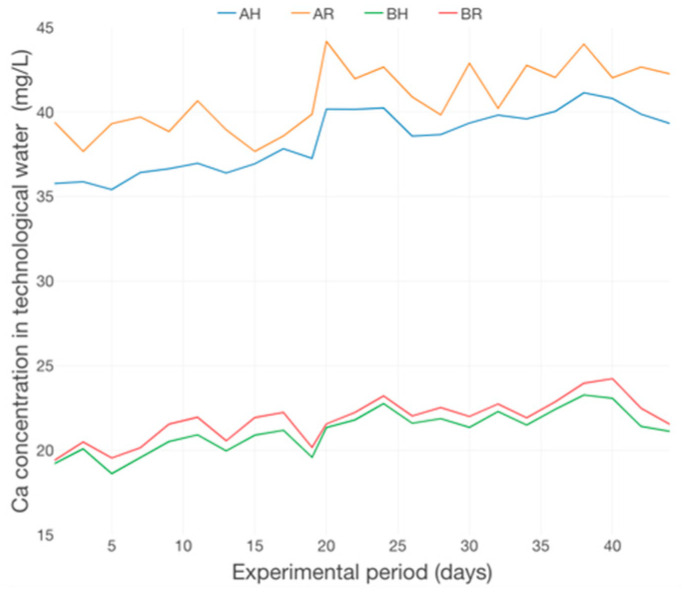
The dynamics of Ca concentration in technological water for each of the experimental variants.

**Figure 24:**
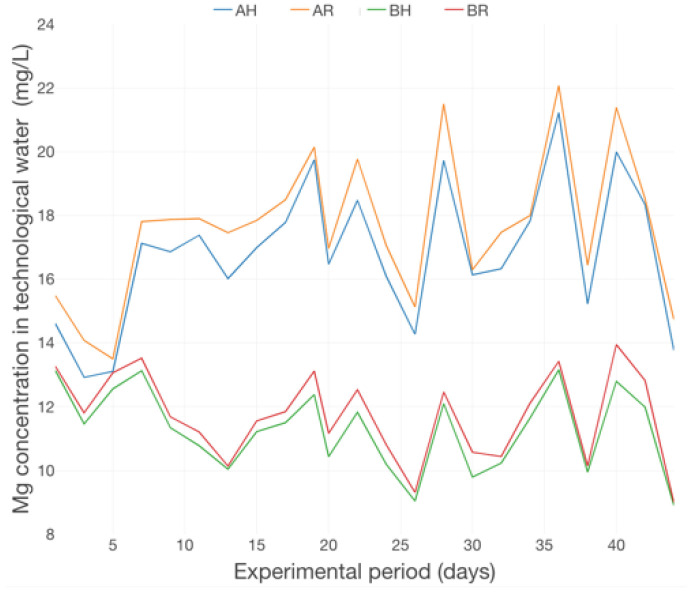
The dynamics of Mg concentration in technological water for each of the experimental variants.

The dynamics of Ca concertation (Figure 23) revealed a slow accumulation trend, manifested especially in the first 3 weeks of the basil production cycle. Also, the Ca concentrations at R GM are superior to those at H GM and reveal multiple peaks in the 2nd period of the production cycle. The dynamics of Mg concentration in water (Figure 24) revealed a relatively constant trend with multiple peaks throughout the experimental period. However, the Mg concentration confirms the significant impact of GM on nutrient dynamics within an aquaponic system, a fact revealed also by other authors [47,49,50].

**Figure 25:**
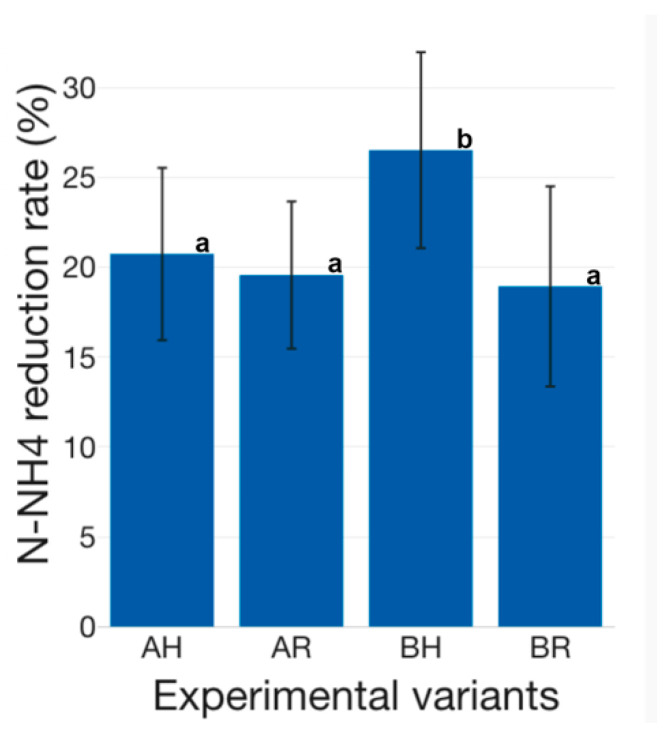
The N-NH_4_ reduction rate and standard error (SE) for each of the experimental variants (Tukey test)—different letters reveal significant statistical differences (*p* < 0.05), whereas the same letter reveal not significant statistical differences (*p* > 0.05).

**Figure 26:**
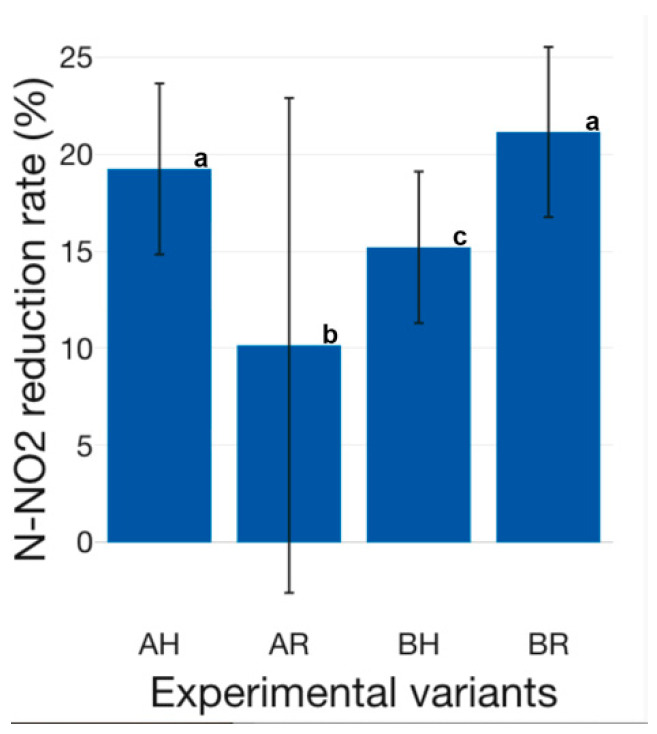
The N-NO_2_ reduction rate and SE for each of the experimental variants (Tukey test)—different letters reveal significant statistical differences (*p* < 0.05), whereas the same letter reveal not significant statistical differences (*p* > 0.05).

**Figure 27:**
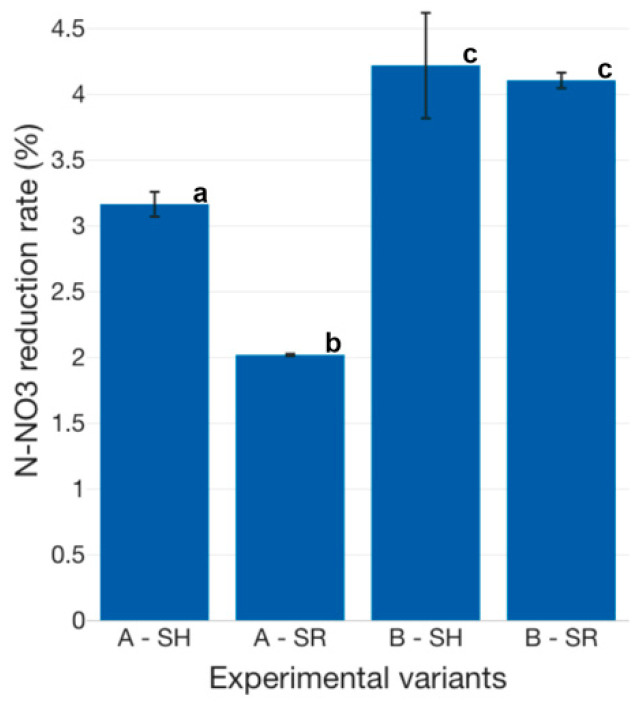
The N-NO_3_ reduction rate and SE for each of the experimental variants (Tukey test)—different letters reveal significant statistical differences (*p* < 0.05), whereas the same letter reveal not significant statistical differences (*p* > 0.05).

The nitrogen compounds reduction capacity is superior if conventional H GM is used, compared to R GM (Figure 25, Figure 26 and Figure 27). Therefore, in the case of N-NH_4_, it can be observed a 20.75 ± 11.10% reduction in AH experimental variant, compared to 19.58 ± 9.47% in AR, while in BH a 26.53 ± 12.62% N-NH_4_ reduction is recorded, compared to 18.95 ± 12.88% in BR (Figure 25). Statistically significant differences (*p* < 0.05) were recorded between BH and the rest of the experimental variants. The results reveal that the H GM performed better in both technological scenarios (A and B), compared to R GM. However, the R GM assures similar N-NH_4_ reduction in both tested technological scenarios (A and B), while H GM N-NH_4_ reduction performance decreases as the amount of fish feed inputs increases. Therefore, if high-intensity sturgeon rearing technologies are targeted, with superior feed inputs compared to those tested in the present study, the R GM could be a solution if N-NH_4_ reduction is one of the desiderata. The NO_2_ reduction rate reveals superior results for H GM, if used in high fish feeding input technological scenarios, as in the case of A (19.23 ± 10.19% at AH, compared to 10.15 ± 29.47 at AR) (Figure 26). However, in low fish feed input technological scenarios, similar to B, the situation is reversing and reveals that R GM manages to perform better, compared to H GM (21.14 ± 10.14% at BR, compared to 15.20 ± 9.04% at BH) (Figure 26). Statistically significant differences (*p* < 0.05) were recorded between all experimental variants, except between AH and BR.

The N-NO_3_ reduction rate is lower, in the case of all experimental variants, compared to N-NH_4_ and N-NO_2_ (Figure 25, Figure 26 and Figure 27). Thus, previous studies concluded that, in the case of basil, plant growth is improved by lower N-NH_4_ exposure, as well as a faster supply of N-NO_3_ as an N source [70]. In aquaponics systems, the crops GM plays a dual role, both as a biofilter, but also as a support media for promoting plant growth. Therefore, the lower reduction rate of N-NO_3_ could be due to the production of supplementary N-NO_3_, during the nitrification process, at the level of GM and, furthermore, the assimilation of the produced N-NO_3_ by the basil biomass. Thus, in addition to the produced N-NO_3_ as a result of the nitrification process at the level of GM, the basil biomass managed to assure an average N-NO_3_ reduction rate of 3.16 ± 0.22% at AH, 2.02 ± 0.02% at AR, 4.22 ± 0.93% at BH and 4.11 ± 0.14% at BR, respectively (Figure 27).

It can be stated that higher N-NO_3_ reduction rates are recorded when less intensive fish-rearing technologies, which require lower feed inputs, are applied, as B technological scenario. This may be due to the higher N-NH_4_ concentration of technological water, reported in the A experimental variants, compared to B variants, since this could inhibit the basil absorption of N-NO_3_. However, some authors [71] concluded that a very low, continuous supply of N-NH_4_ can be of great importance in balancing anions and cations absorbed by the plant. Therefore, controlling the N-NH_4_ concentration within aquaponics systems could be one of the main keys towards increasing the sustainability and nutrient efficiency of multi-trophic aquaculture. Statistically significant differences (*p* < 0.05) are recorded between all tested experimental variants, except BH and BR.

Nitrification is a biological process that maintains water quality in quaponic systems by converting a toxic form, ammonia-nitrogen (N-NH_3_), into a non-toxic form, nitrate (N-NO_3_), to fish and plants in biofiltration units. The intermediate product of nitrification, nitrite (N-NO_2_), is also known to be toxic to both fish and plants at low levels.

In our study was a clear tendency of an increased initial concentrations of mineral nutrients (N-NO_3_, Ca, and Mg) but decreased concentrations of other compounds (N-NO_2_, N-NH_4_).

The growth of romaine and iceberg lettuce was reduced by N-NO_2_ at concentrations as low as 5 mg/L in hydroponic solution [72].

Direct contact with nitrite at this concentration can damage root tips as demonstrated in tobacco (*Nicotiana tabacum* L.) [73]. It is well expected that N-NO_2_ concentrations fluctuate more widely in aquaponics than those in hydroponics, especially after feeding, possibly exposing roots to a detrimental level of N-NO_2_ to root growth [64].

In the same time a higher level of N-NO_2_ may be involved in reduced root growth in aquaponics, subsequently affecting crop yield and quality [64].

Regarding to N-NO_3_ concentration, some studies have shown that under pH 6.0, the N-NO_3_ concentration even dropped a little because of enhanced plant growth [74].

This might because nitrification was inhibited at low pH [75]. This happens due to the fact that more nitrogen loss occurred at pH 7.5 and 9.0. Under pH 7.5, more N-NO_3_ was consumed by denitrification, and under pH 9.0, production of N-NO_3_ decreased because of more N-NH_3_ evaporation [74].

In our case, towards the end of the experimental period, the N-NO_3_ concentration registered an increase, which means that more plants can be supported after increasing the nitrogen supply. Therefore, the ratio of suitable plants/fish was needed for long-term aquaponics to control nutrient levels.

### 2.4. Prediction Models for the Development of Black Box Soft Sensors, Targeting Main Water Quality Parameters

The development of black box soft sensors mainly targets the use of multiple linear regression in order to predict nitrogen compounds concentrations since those are the most important among high-cost-demanding parameters which should be considered to be real-time monitored. However, since not all soft sensors are based on linear models, the analytical framework developed in the present study considers generalised additive models (GAMs) as an adaptation, in order to deal with non-linear data, while maintaining explainability. In the end, the applied models should be able to identify strong existing data patterns, formalized as non-linear predictive models, validated by their high accuracy in predicting previously unseen data samples.

#### 2.4.1. The Correlation Matrix

As already stated in a previous study [76], the correlation matrix is used as a tool to summarize the linear relationships (MLR) existent in the database, as well as for identifying strong and relevant relationships that could be further modelled in order to develop soft sensors. The correlation matrixes display the Pearson correlation coefficients between all the available variables—if the correlation coefficient between two variables is +/−0.8, then this reveals a strong positive/negative linear correlation between the two variables [77].

**Figure A13:**
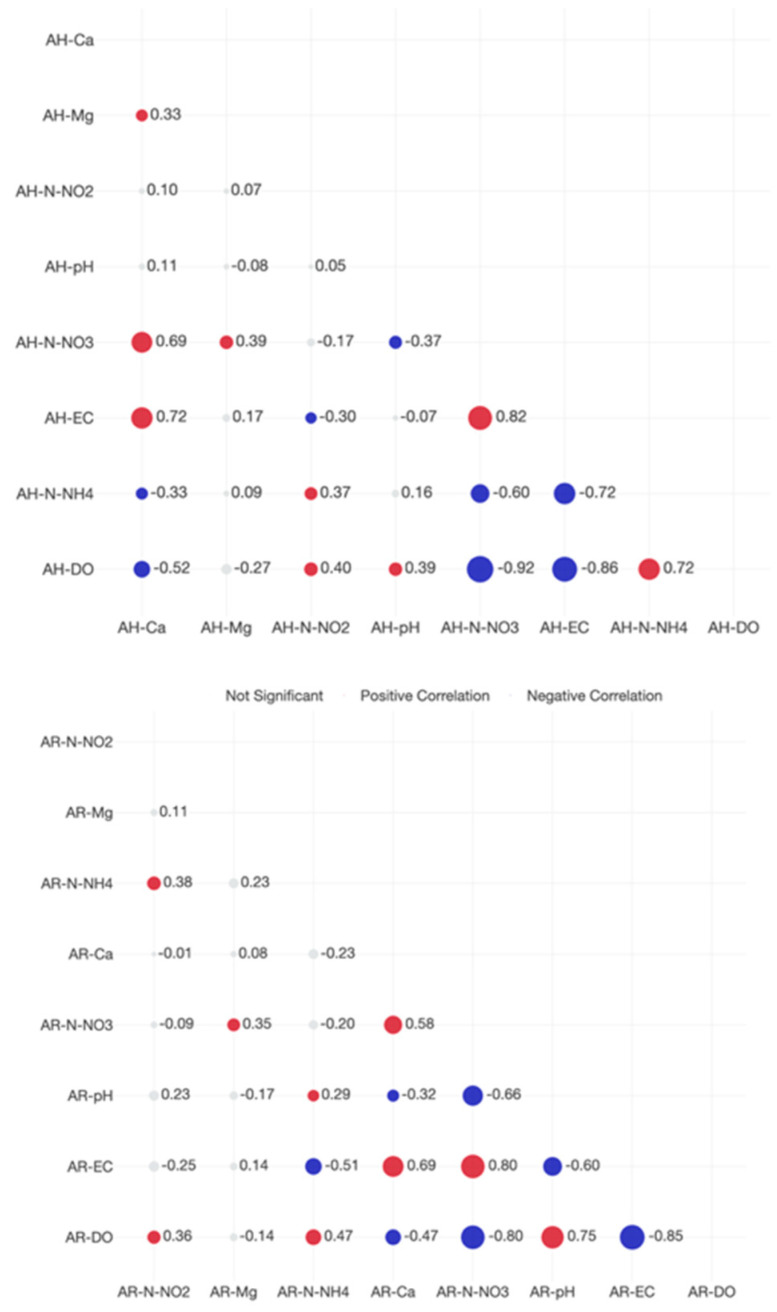

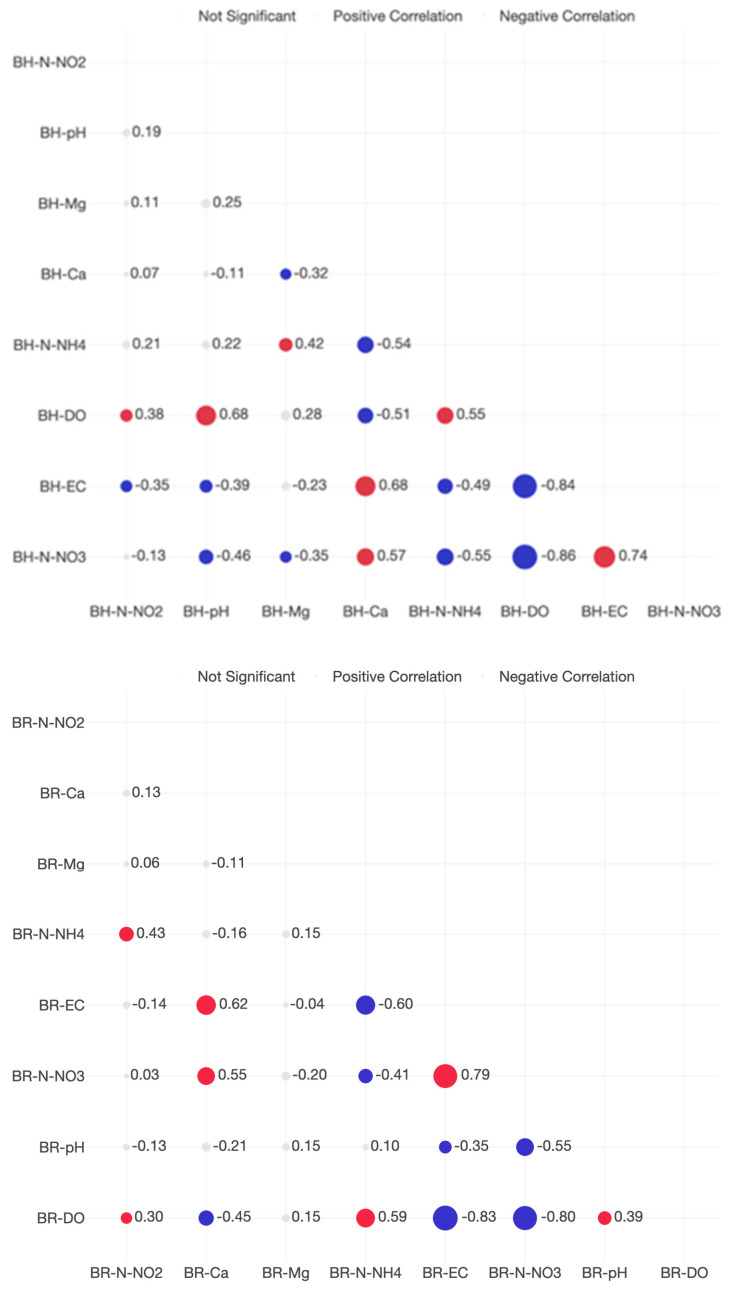
The correlation matrices of water quality parameters for each of the experimental variants.

The correlation matrix for all experimental variants reveals strong negative correlations between N-NO_3_–DO and DO–EC, respectively, while strong positive correlations are recorded between N-NO_3_-EC (Figure A13—Appendix B). Thus, 3 linear relations are identified, a finding which is used in order to reduce the linear model’s multicollinearity issue.

#### 2.4.2. The MLR Prediction Models

The MLR models proposed for developing the nitrogen compounds soft sensors are presented in Table 6. It can be revealed that the models addressed to the prediction of N-NO_3_ recorded the highest degree of precision since each of them explains more than 80% of the dependent variable variance (Table 6). The lowest prediction performance is generally associated with MLR models which target the determination of N-NO_2_ concentration in the technological water since they explain between 26.8% and 50.5% of the dependent variable variance (Table 6). Also, the N-NH_4_ prediction models record good predictivity accuracy, especially in the case of AH, where they explain 64.4% of the dependent variable variance, followed by BR (54.3%) (Table 6).

The MLR models reveal that for N-NH_4_ prediction, the N-NO_2_ concentration and the DO are the most important independent variables, followed by pH, especially in the case of variants part of low fish feed input (B) technological scenario (Table 6). Also, The N-NO_2_ MLR models mostly rely on DO and N-NH_4_ as independent variables, as well as pH in the case of AH, BR and BH, while N-NO_3_ models are considered the most complex since they imply a larger number of significant independent variables as DO, N-NO_2_, N-NH_4_, pH and even Ca, in the case of AH (Table 6).

#### 2.4.3. The Generalized Additive Models (GAM) for Developing Black-Box Soft Sensors

In order to cover the non-linear data, GAMs were used, especially since, according to a previous study [78], a big advantage of these models stems from their interpretability, the contribution of each predictor being clearly presented considering that the outcome is revealed as a sum of arbitrary functions of each feature by replacing the beta coefficients from linear regression, with flexible functions (splines) that allow for nonlinear relationships to be modelled.

**Figure A14:**
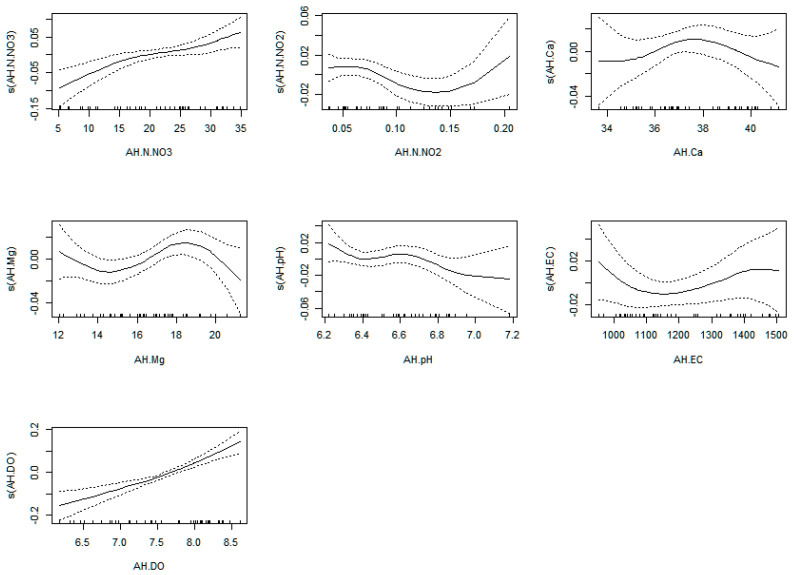
The GAM predictors influence over N-NH_4_ for AH experimental variant.

Thus, the GAM for predicting N-NH_4_ in the case of the AH experimental variant revealed high accuracy metrics (Rsq. = 0.914, Adj Rsq. = 0.895), emphasizing the continuous upward trend as the N-NO_3_ is increasing (Figure A14, Appendix C). Also, considering the same experimental variant (AH), the N-NH_4_ reveal a decreasing trend as N-NO_2_ starts to increase (until the concentration of 0.1 mg/L), followed by a downward tendency, mostly associated with the stability status of the aquaponics systems (concentrations of N-NO_2_ of 0.13–0.15 mg/L) and a fast upward trend if N-NO_2_ concentration passes the threshold of 0.15 mg/L (Figure A14, Appendix C). The Ca concentration between 36–40 mg/L and Mg concentrations between 18–20 mg/L are associated with the highest N-NH_4_ predicted concentrations in the case of the AH variant, while if pH is considered as an independent variable, its increase, especially over 6.8 upH, will be associated with a decrease of N-NH_4_. The DO strong and rapid increase predicts the increase of N-NH_4_ concentration, a fact valid if EC ranges between 1150 and 11,350 μs/cm.

**Figure A15:**
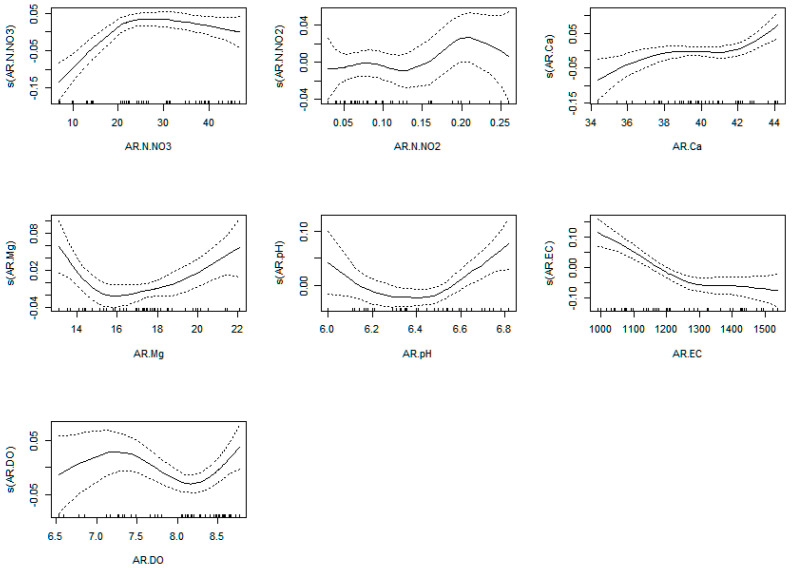
The GAM predictors influence over N-NH_4_ for AR experimental variant.

The GAM for predicting N-NH_4_ in the case of the AR experimental variant revealed high accuracy metrics (Rsq. = 0.918, Adj Rsq. = 0.900), emphasizing the continuous strong upward trend as the N-NO_3_ is increasing to 20 mg/L, followed by an equilibrium status (Figure A14, Appendix C). Also, considering the same experimental variant (AR), the N-NH_4_ reveal an increasing trend as N-NO_2_ starts to increase between 0.15–0.20 mg/L, followed by a decrease if the concentration overcomes 0.20 mg/L (Figure A15, Appendix C). The Ca upward trend is associated, in the case of AR, with an increase of N-NH_4_ concentration, similar to Mg if it overcomes 16 mg/L concentration in the technological water. The pH dynamics divides the N-NH_4_ prediction trends into a decreasing segment, if pH decreases from 6 to 6.5 upH, followed by an increasing tendency, until pH reaches 6.8 upH (Figure A15, Appendix C). The EC increasing trend predicts a downward tendency of N-NH_4_ concentration in water, at AR, while if considering DO as an independent variable, the N-NH_4_ is predicted to decrease if DO ranges between 7.6–8.3 upH Figure A15, Appendix C).

**Figure A16:**
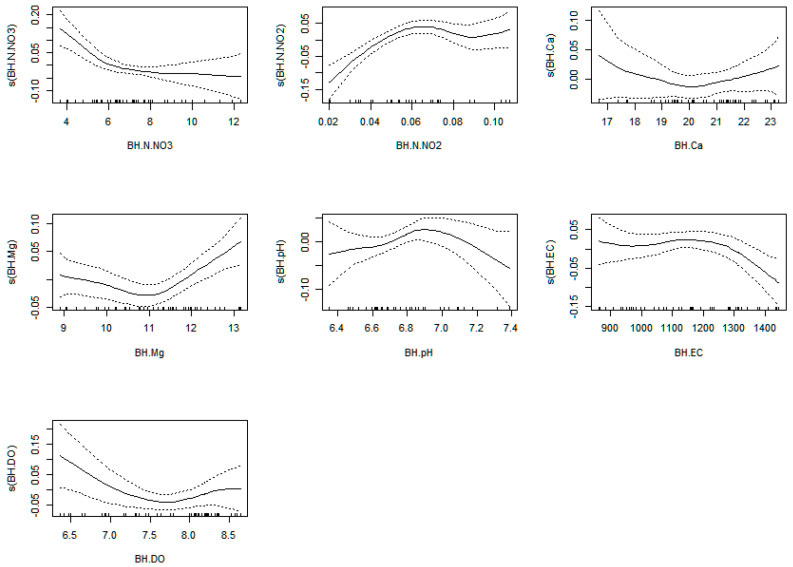
The GAM predictors influence over N-NH_4_ for BH experimental variant.

The GAM for predicting N-NH_4_ in the case of the BH experimental variant revealed high accuracy metrics (Rsq. = 0.876, Adj Rsq. = 0.849), although lower compared to both AR and AH variants, emphasizing the continuous strong upward trend as the N-NO_3_ decreases under 6 mg/L (Figure A16, Appendix C). Also, considering the same experimental variant (BH), the N-NH_4_ reveal an increasing trend as N-NO_2_ starts to increase up to 0.07 mg/L (Figure A16, Appendix C). The Ca concentration of 20 mg/L and Mg concentration of 10.6 mg/L are associated with the lowest N-NH_4_ concentration but, either an increase or decrease starting from the above-mentioned points will generate significant upward trends of the dependent variable (Figure A16, Appendix C). The pH and EC dynamics predict a decrease in N-NH_4_ concentration if the values exceed 6.9 upH and 1270 μs/cm, respectively. However, the DO decrease below 7.6 mg/L will predict a strong increase oh N-NH_4_ concentration in water, at BH (Figure A16, Appendix C).

**Figure A17:**
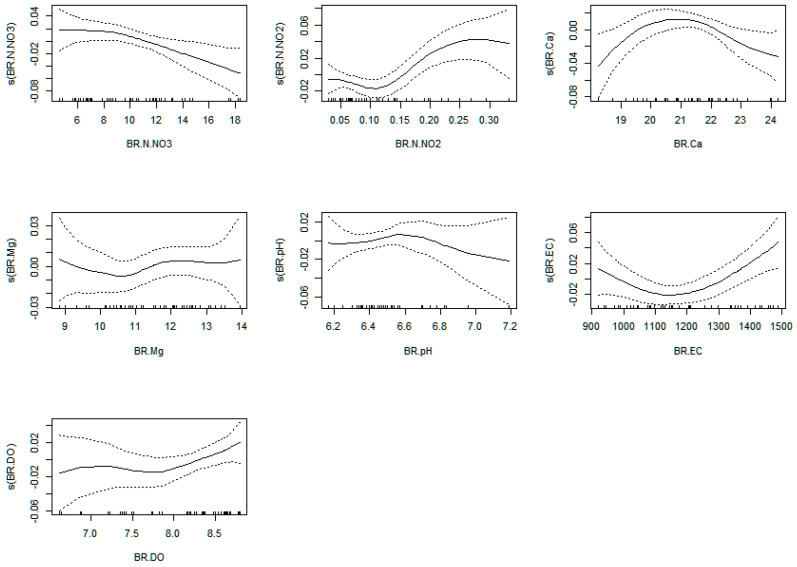
The GAM predictors influence over N-NH_4_ for BR experimental variant.

The GAM for predicting N-NH_4_ in the case of the BR experimental variant revealed high accuracy metrics (Rsq. = 0.849, Adj Rsq. = 0.816), similar to those recorded at BH and lower compared to both AR and AH variants, emphasizing the continuous strong upward trend as the N-NO_3_ decreases (Figure A17, Appendix C). Also, considering the same experimental variant (BR), the N-NH_4_ reveal an increasing trend as N-NO_2_ starts to increase from 0.11 mg/L (Figure A17, Appendix C). The Ca concentration of 21 mg/L, Mg concentration ranging from 12 to 14 mg/L and pH of 6.55 upH are associated with the highest N-NH_4_ concentration at BR (Figure A17, Appendix C). The EC and DO dynamics predict an increase in N-NH_4_ concentration if the values exceed 1200 μs/cm and 7.9 mg/L, respectively (Figure A17, Appendix C).

**Figure A18:**
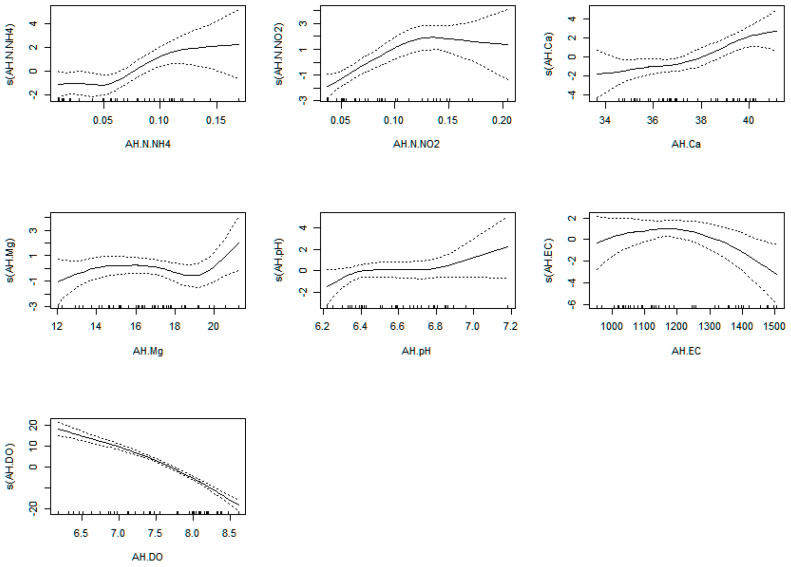
The GAM predictors influence over N-NO_3_ for AH experimental variant.

The GAM for predicting N-NO_3_ in the case of the AH experimental variant revealed high accuracy metrics (Rsq. = 0.991, Adj Rsq. = 0.998), emphasizing the continuous strong upward trend as the N-NH_4_ increases (Figure A18, Appendix C). Also, considering the same experimental variant (AH), the N-NO_3_ reveal an increasing trend as N-NO_2_ starts to increase up to 0.14 mg/L (Figure A18, Appendix C). The Ca concentration increasing trend reveals as similar trend for N-NO_3_ prediction, while the Mg concentrations over 18.5 mg/L and pH over 6.8 upH are also predicting a fast-increasing trend for the dependent variable (Figure A18, Appendix C). The EC increase over 1200 μs/cm predicts a decrease of N-NO_3_, while the DO increase also predicts the decrease of the dependent variable at the AH experimental variant (Figure A18, Appendix C).

**Figure A19:**
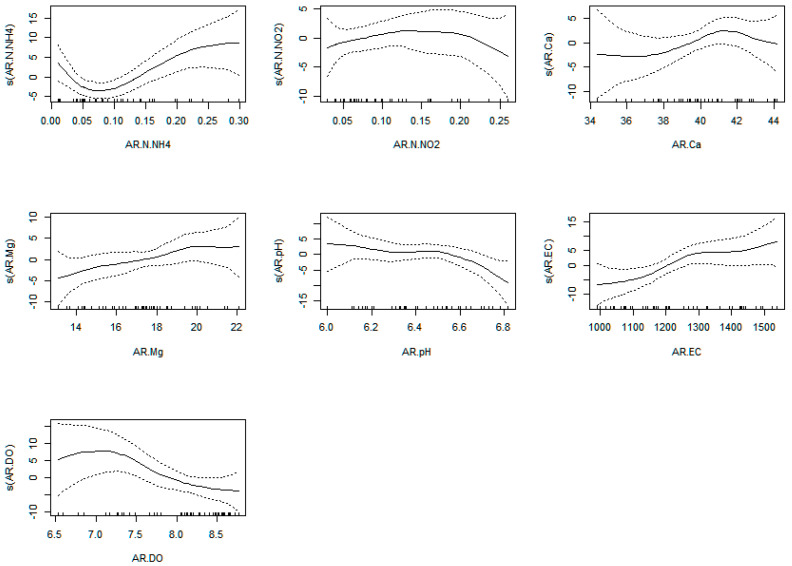
The GAM predictors influence over N-NO_3_ for AR experimental variant.

The GAM for predicting N-NO_3_ in the case of the AR experimental variant revealed high accuracy metrics (Rsq. = 0.930, Adj Rsq. = 0.915), emphasizing the continuous strong upward trend as the N-NH_4_ increases in concentration, over 0.10 mg/L (Figure A19, Appendix C). Also, considering the same experimental variant (AR), the N-NO_3_ reveal a decreasing trend as N-NO_2_ starts to increase over 0.21 mg/L (Figure A19, Appendix C). The Ca concentration between 37–41 mg/L generates an increase in N-NO_3_ prediction, while the Mg concentrations over 17 mg/L and pH between 6–6.4 upH are also predicting the maximum concentration points of the dependent variable (Figure A19, Appendix C). The EC increase over 1150 μs/cm and DO decrease are both predicting an increase of N-NO_3_ at the AH experimental variant (Figure A19, Appendix C).

**Figure A20:**
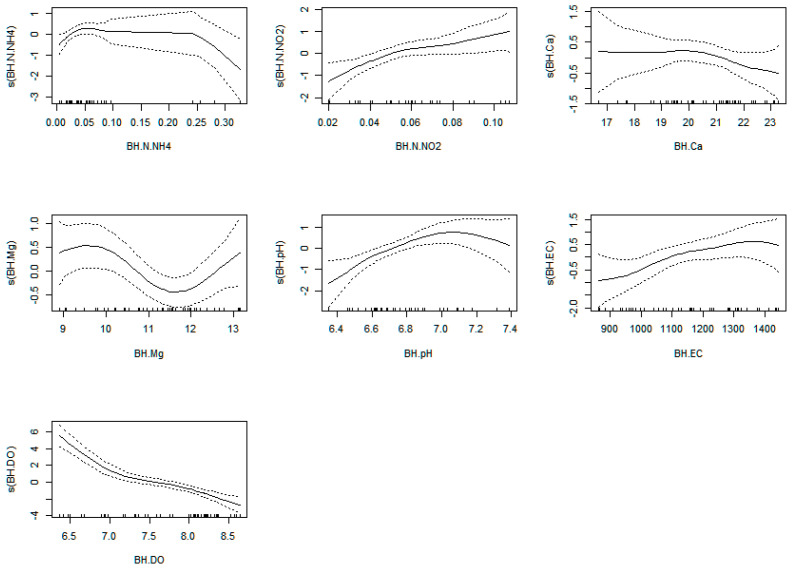
The GAM predictors influence over N-NO_3_ for BH experimental variant.

The GAM for predicting N-NO_3_ in the case of the BH experimental variant revealed high accuracy metrics (Rsq. = 0.956, Adj Rsq. = 0.947), emphasizing a strong downward trend as the N-NH_4_ increases in concentration, over 0.25 mg/L (Figure A20, Appendix C). Also, considering the same experimental variant (BH), the N-NO_3_ reveal an increasing trend as N-NO_2_ increases (Figure A20, Appendix C). The increase of Ca concentration over 19.5 mg/L predicts a decrease of N-NO_3_, while the Mg concentrations between 11–12 mg/L are associated with the lowest values recorded for the dependent variable (Figure A20, Appendix C). Both the pH increases up to 7 upH and the EC increase over 950 μs/cm are also predicting the increase of N-NO_3_ concentration at the BH experimental variant (Figure A20, Appendix C). However, in the case of BH, the DO decrease generates a decreasing trend of N-NO_3_ concentration in technological water (Figure A20, Appendix C).

**Figure A21:**
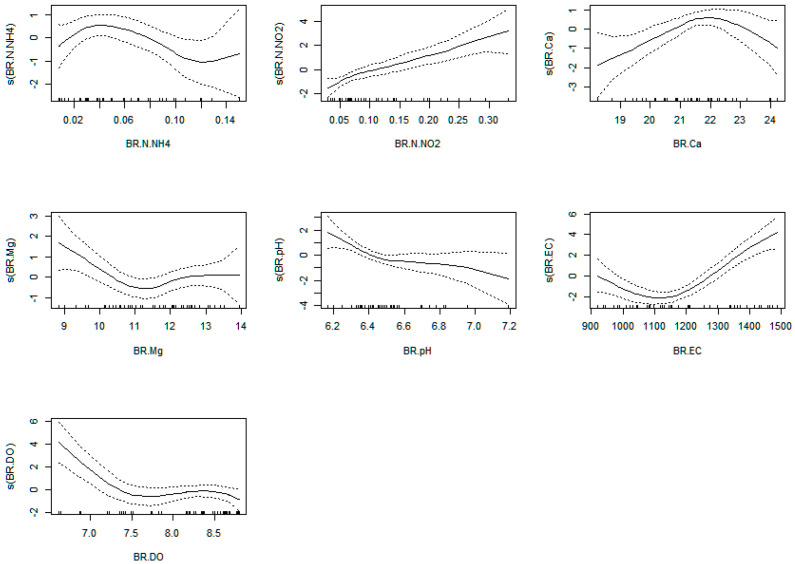
The GAM predictors influence over N-NO_3_ for BR experimental variant.

The GAM for predicting N-NO_3_ in the case of the BR experimental variant revealed high accuracy metrics (Rsq. = 0.970, Adj Rsq. = 0.964), emphasizing a strong downward trend as the N-NH_4_ ranges between 0.01–0.04 mg/L (Figure A21, Appendix C). Also, considering the same experimental variant (BR), the N-NO_3_ reveal an increasing trend as N-NO_2_ increases (Figure A21, Appendix C). The increase of Ca concentration up to 22 mg/L predicts an increase of N-NO_3_, while the Mg concentrations of 9–9.5 mg/L, pH of 6.2–6.3 upH and DO of 6.5–6.6 mg/L are associated with the highest values recorded for the dependent variable (Figure A21, Appendix C). The EC increase over 1150 μs/cm is also predicting the increase of N-NO_3_ concentration at the BR experimental variant (Figure A21, Appendix C). 

**Figure A22:**
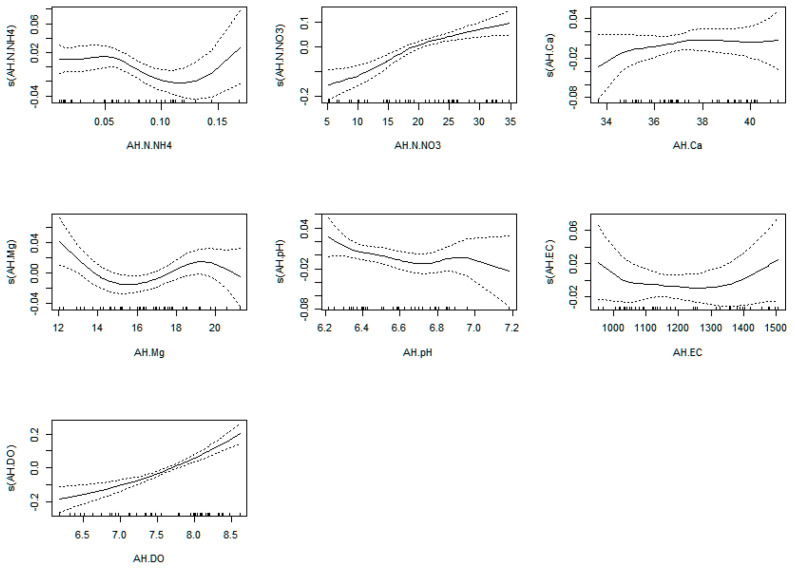
The GAM predictors influence over N-NO_2_ for AH experimental variant.

The GAM for predicting N-NO_2_ in the case of the AH experimental variant revealed high accuracy metrics (Rsq. = 0.869, Adj Rsq. = 0.841), emphasizing a strong downward trend as the N-NH_4_ ranges between 0.06–0.13 mg/L (Figure A22, Appendix C). Also, considering the same experimental variant (AH), the N-NO_2_ reveal an increasing trend as N-NO_3_ increases (Figure A22, Appendix C). The increase of Ca concentration between 34–35 mg/L predicts an increase of N-NO_2_, while the Mg concentrations of 12–14 mg/L, pH of 6.2–6.3 upH and EC of 950–1050 μs/cm predict a significant decrease of the dependent variable (Figure A22, Appendix C). The DO increase predicts an increase of N-NO_2_ concentration at the AH experimental variant (Figure A22, Appendix C).

**Figure A23:**
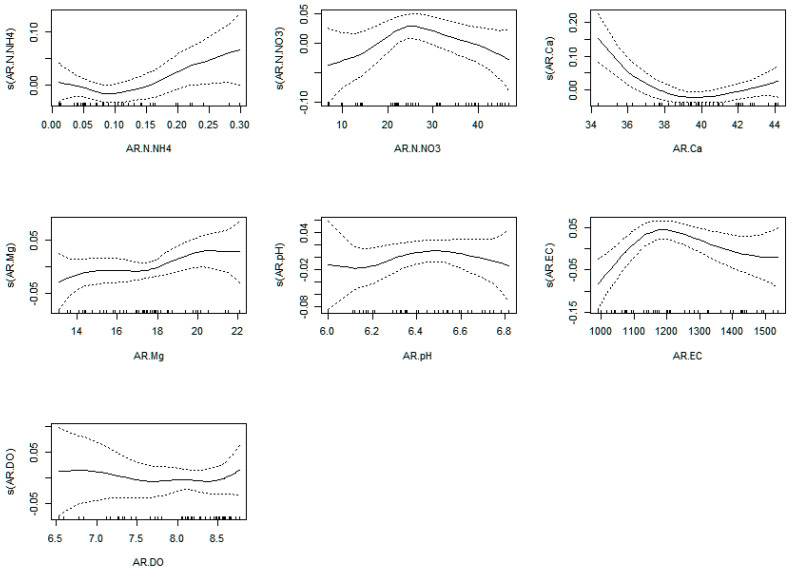
The GAM predictors influence over N-NO_2_ for AR experimental variant.

The GAM for predicting N-NO_2_ in the case of the AR experimental variant revealed high accuracy metrics (Rsq. = 0.869, Adj Rsq. = 0.841), emphasizing a strong upward trend as the N-NH_4_ increase over 0.10 mg/L (Figure A23, Appendix C). Also, considering the same experimental variant (AR), the N-NO_2_ records a maximum peak as N-NO_3_ reaches values between 25–28 mg/L (Figure A23, Appendix C). The Ca concentration between 34–35 mg/L predicts an increase of N-NO_2_, similar to the Mg concentrations over 18 mg/L (Figure A23, Appendix C). The pH of 6.2–6.8 upH and EC of 980–1180 μs/cm predict high concentrations of the dependent variable (Figure A23, Appendix C). The DO increase over 8.3 mg/L predicts an increase of N-NO_2_ concentration at the AR experimental variant (Figure A23, Appendix C).

**Figure A24:**
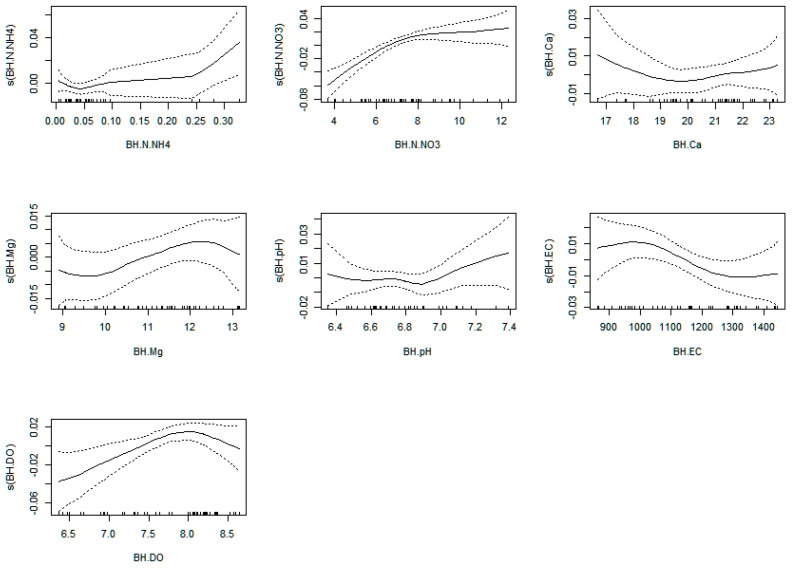
The GAM predictors influence over N-NO_2_ for BH experimental variant.

The GAM for predicting N-NO_2_ in the case of the BH experimental variant revealed high accuracy metrics (Rsq. = 0.859, Adj Rsq. = 0.829), emphasizing a strong upward trend as the N-NH_4_ increase over 0.24 mg/L (Figure A24, Appendix C). Also, considering the same experimental variant (BH), the N-NO_2_ concentration increase as N-NO_3_ reaches values between 4–9 mg/L (Figure A24, Appendix C). The Ca concentration between 17–20 mg/L predicts an increase of N-NO_2_, a trend predicted also by the Mg concentrations between 9.8–12.4 mg/L and by the pH values over 6.9 upH (Figure A24, Appendix C). However, low DO concentrations, under 7.6 mg/L and EC between 1100–1230 μs/cm predict low N-NO_2_ concentrations in the technological water (Figure A24, Appendix C).

**Figure A25:**
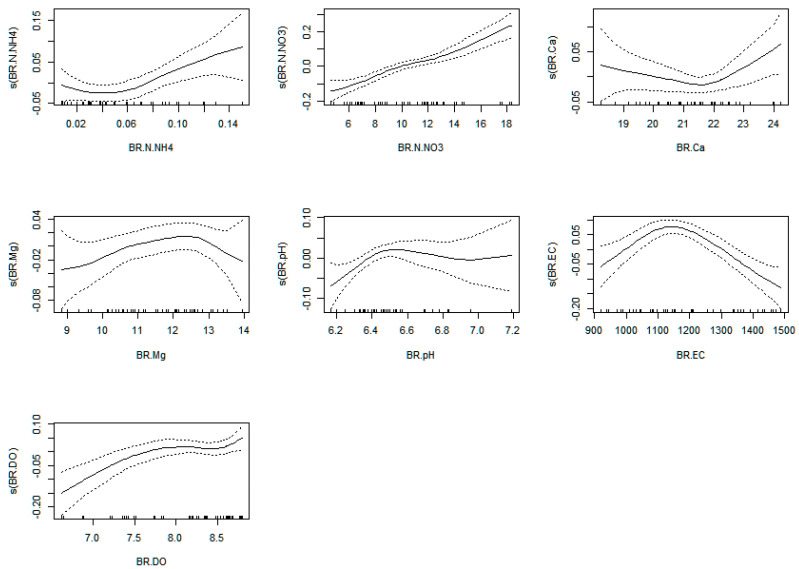
The GAM predictors influence over N-NO_2_ for BR experimental variant.

The GAM for predicting N-NO_2_ in the case of the BR experimental variant revealed high accuracy metrics (Rsq. = 0.874, Adj Rsq. = 0.847), emphasizing a strong upward trend as the N-NH_4_ increase over 0.06 mg/L (Figure A25, Appendix C). Also, considering the same experimental variant (BR), the N-NO_2_ concentration increase as N-NO_3_ increase (Figure A25, Appendix C). The Ca concentration between 21.4–21.8 mg/L predicts the lowest N-NO_2_ concentrations (Figure A25, Appendix C). However, an increase in Mg up to 12.8 mg/L, an increase of pH up to 6.5 upH, of EC up to 1180 μs/cm and DO up to over 8.5 mg/L predicts an increase of N-NO_2_ within BR experimental variant (Figure A25, Appendix C).

The GAM results confirm a previous study [79] which emphasizes that values of specific N-NO_3_ oxidation rate at low N-NH_4_ are considerable higher, suggesting that nitrification at high N-NH_4_ levels will invariably result in N-NO_3_ accumulation. Also, the results confirmed previous findings [79] according to which low oxygen tensions will exacerbate nitrite accumulation.

#### 2.4.4. The Principal Component Analysis (PCA) of Water Quality Parameters

**Figure 28:**
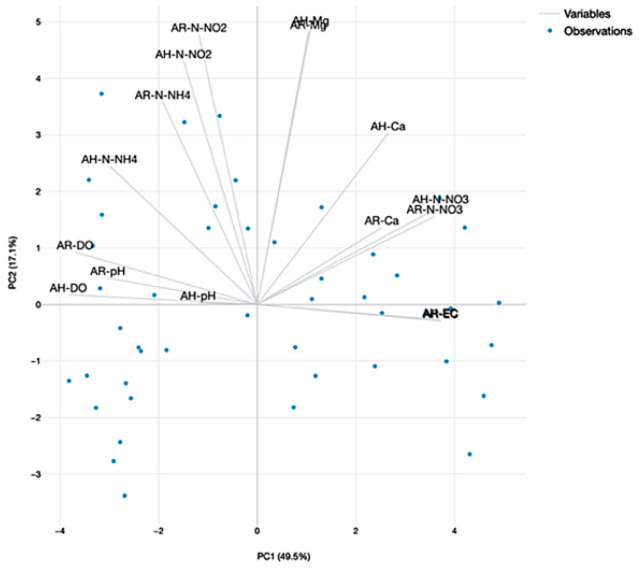
The PCA for water quality parameters associated to A dataset.

**Figure 29:**
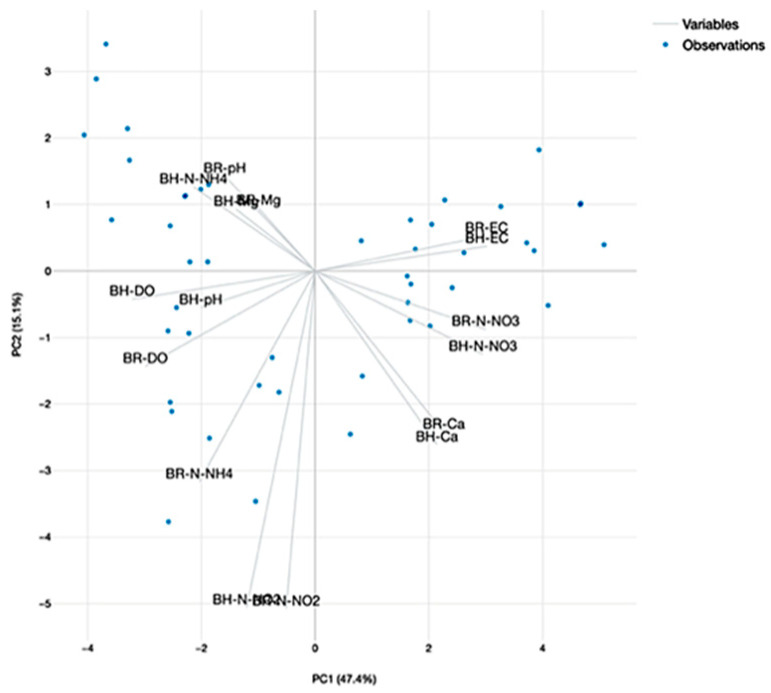
The PCA for water quality parameters associated to B dataset.

The PCA analysis revealed two major components, with an eigenvalue greater than 1, which manage to explain more than 66.6% of data variance in the dataset that includes the experimental variants involved in A technological scenario (Figure 28) and 62.5% for the dataset associated to B technological scenario (Figure 29). It can be stated that in the case of A dataset, the pH and DO are highly correlated, both at R and H GM variants and are integrated into the first component, which explain 49.5% of the variance (Figure 28). However, the PCA of the B dataset reveal also high correlations between DO and pH for H GM experimental variant and are integrated into first component which explain 47.4% of the variance, while N-NH_4_ and Mg for H GM are correlated and integrated in the second component which explain 15.1% of the variance (Figure 29).

### 2.5. Quality Analysis of the Resulting Basil Biomass

Aquaponics systems have, as one of the main advantages, the possibility of controlling the quality of crop production since water quality significantly impacts the quality of final products [80,81]. As an example, an increase in salinity, sugar, organic acids, amino acids, K and Mg improves the organoleptic features of soilless-grown plants and enhances the production of compounds providing numerous health benefits (polyphenols, carotenoids, vitamin C) [82,83].

**Figure 30:**
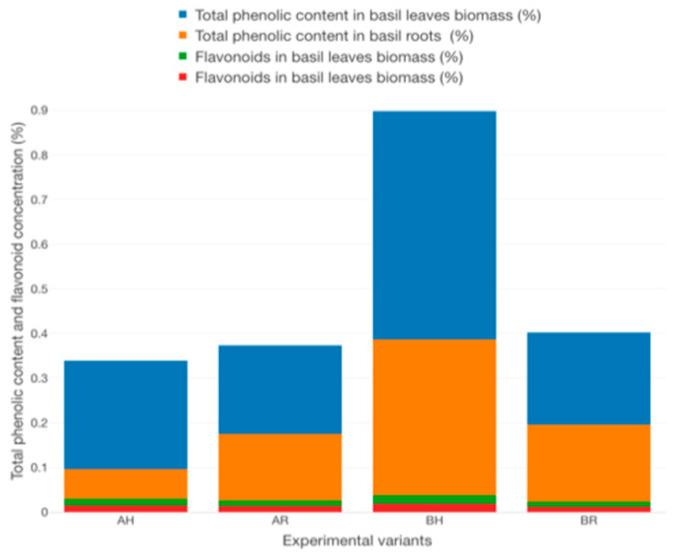
The total phenolic content and flavonoids concentration in basil roots and leaves biomass growth in each of the experimental variants.

In order to reveal the impact of GM on the quality of aquaponic cultured basil, considering different technological scenarios, the total phenolic content, flavonoid concentration and DPPH scavenging activity were evaluated from both roots and leaves biomass. Therefore, in terms of total phenolic content, the basil leaves biomass registered higher average values (0.243 ± 0.011% at AH, 0.199 ± 0.005% at AR, 0.512 ± 0.006% at BH and 0.207 ± 0.012% at BR) compared to basil roots (0.066 ± 0.001% at AH, 0.149 ± 0.002% at AR, 0.348 ± 0.003% at BH and 0.172 ± 0.001% at BR) (Figure 30). Statistically significant differences (*p* < 0.05) are recorded between all the experimental variants (Tukey test). However, in terms of flavonoids content, the basil root recorded superior values (0.015 ± 0.001% at AH, 0.013 ± 0.001% at AR, 0.019 ± 0.001% at BH and 0.012 ± 0.002% at BR), compared to basil leaves biomass (0.124 ± 0.001% at AH, 0.150 ± 0.001% at AR, 0.169 ± 0.002% at BH and 0.111 ± 0.001% at BR) (Figure 30). Statistically significant differences (*p* < 0.05) are recorded between AR and the rest of the experimental variants in terms of flavonoids content from basil roots, and between all experimental variants in terms of basil leaves flavonoids content (Tukey test).

**Figure 31:**
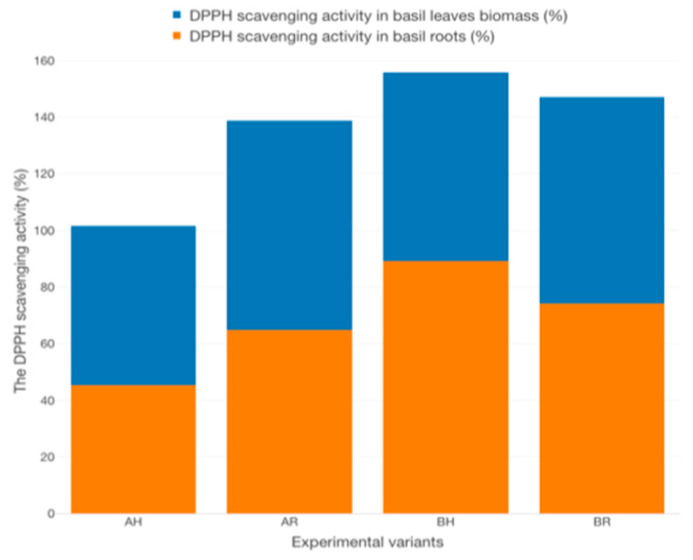
The DPPH scavenging activity in basil roots and leaves biomass growth in each of the experimental variants.

The DPPH scavenging activity in basil leaves recorded the highest value at AR (73.96 ± 1.08%), followed by BR (72.78 ± 0.30%), BH (66.73 ± 0.53%) and AH (56.25 ± 1.06%) (Figure 31). However, in basil roots, the highest DPPH scavenging activity was found at BH (89.15 ± 0.92%), followed by BR (74.2 ± 0.98%), AR (64.73 ± 1.24%) and AH (45.35 ± 0.55%). Statistically significant differences (*p* < 0.05) are recorded between all the experimental variants (Tukey test).

The high concentration in total phenolics and flavonoids, recorded at BH could be related to Fe concentration in water, since this element was considered the most limited within the water matrix elements, due to its low concentrations during the experimental period. Previous studies also reported that Fe limitation increases the production of polyphenols and flavonoids as well as the antioxidant capacity in crops since Fe deficiency stimulates phenylalanine ammonia-lyase, an enzyme that catalyses the conversion of *L*-phenylalanine into *trans*-cinnamic acid, which plays a key role in the biosynthesis of phenolic compounds [84]. A limited number of studies evaluated the content of various phytochemicals and antioxidant activity of plants grown aquaponically, in different conditions. Some authors reported a reduced total phenolic content and antioxidant capacity of the leaves of *Ipomoea batatas* cultured aquaponically, in comparison with plants grown in the soil (200 vs. 345 μg total phenols/g dry weight (DW), 9685.1 vs. 17,619.6 μg TEAC/g DW, where TEAC represents trolox equivalent antioxidant capacity) [85]. Other authors found no significant differences in total phenolic and flavonoid contents between creole tomatoes (*Solanum lycopersicum* and *S. pimpinellifolium*) grown aquaponically and those grown on organic soil, whereas the antioxidant activity was higher in the former [86].

Other studies reported a total phenolics concentration of 1.02 mg gallic acid equivalents/g fresh weight in aquaponically cultured basil (*Ocimum basilicum*) leaves [87]. When reporting to the DW, total phenolics and antioxidant effects in aquaponic basil recorded values of 7.25 mg gallic acid equivalents/g dry weight and 28.04 moles of ascorbic acid equivalent/g DW [87]. Therefore, it can be stated that cultural conditions and water chemical composition considerably influence the biosynthetic capacity of aquaponic plants and explain, in great part, different results obtained for the same plant species. For example, an increase in Mn in the aquaponic system and short-term salinity stress are facile approaches to enhance polyphenols accumulation and antioxidant capacity of aquaponic plants [85].

**Figure 32:**
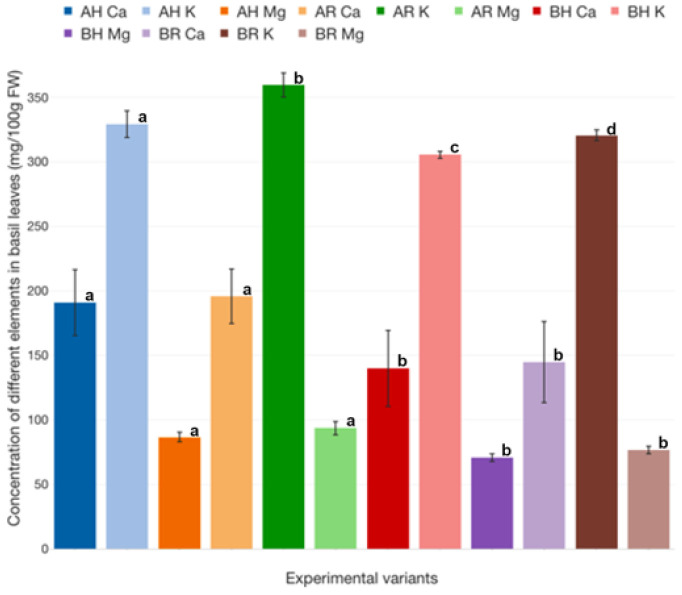
The concentration of different elements (Ca, Mg, K) in basil leaves biomass and SE, growth in each of the experimental variants (Tukey test)—different letters reveal significant statistical differences (*p* < 0.05), whereas the same letter reveal not significant statistical differences (*p* > 0.05).

The highest concentration of Ca is encountered in AR basil leaves (195.73 ± 17.03 mg/100 g FW), while the lowest concentration is associated with BH basil (139.98 ± 23.72 mg/100 g FW). However, statistically significant differences (*p* < 0.05) are recorded only between A and B experimental variants (Figure 32).

However, in the case of K concentration in basil leaves, statistically significant differences (*p* < 0.05) are also reported between AR (359.67 ± 7.58 mg/100 g FW) and AH (329.11 ± 8.27 mg/100 gFW). Also, the highest concentration of Mg confirms the previous pattern, being encountered in AR experimental variant (93.57 ± 4.28 mg/100 g FW), while the lowest is reported for BH (70.72 ± 2.29 mg/100 g FW). Therefore, it can be stated that R GM assures a better valorization of all analyzed elements (Ca, Mg, K) at the level of basil leaves, compared to H GM.

**Figure 33:**
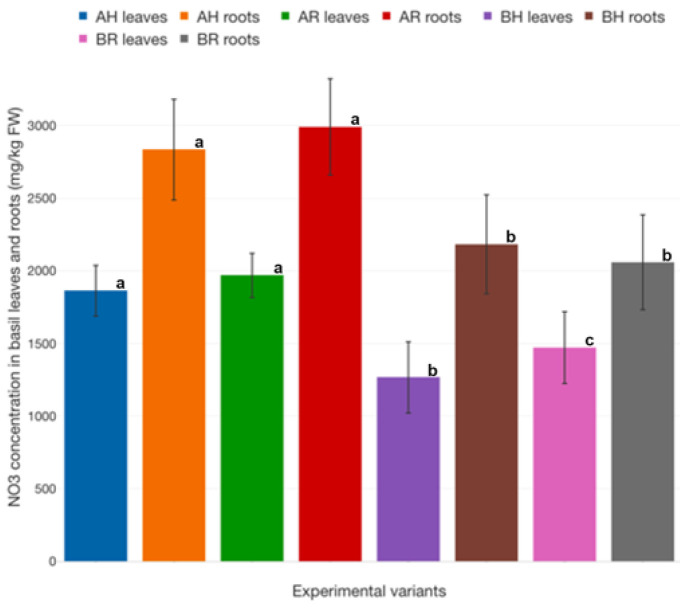
The concentration of NO_3_ in basil roots and leaves biomass and SE, growth in each of the experimental variants (Tukey test)—different letters reveal significant statistical differences (*p* < 0.05), whereas the same letter reveal not significant statistical differences (*p* > 0.05).

The basil roots accumulate NO_3_ in higher concentrations, compared to the leaves (Figure 33). However, the highest NO_3_ accumulation at the level of leaves is recorded at R GM (1970.19 ± 122.75 mg/kg FW at AR and 1863.45 ± 140.23 mg/kg FW at BR), compared to H GM, although no significant differences are recorded between the variants with different GM, part of the same technological scenario (Figure 33). Also, the roots of AR basil recorded the highest NO_3_ concentration (2991.29 ± 267.61 mg/kg FW), while considering the B technological scenario, the R GM records the lower NO_3_ concentration (2057.06 mg/kg FW263.47 mg/kg FW).

Similar to most studies, the design of the current study is subject to limitations. Therefore, the study experimental period was limited to a single basil production cycle. Thus, the impact of new R GM on a long-term usage period is still unknown, considering that during long-term consecutive production cycles, the efficiency of aquaponics GM in terms of nitrification is decreasing, especially due to the accumulation of organic matter. However, the data revealed in current study clearly indicates a lower organic matter accumulation for R GM, compared to conventional H GM.

Also, it is recommended that future studies should consider testing the R GM within other technological scenarios which will imply different fish and plant species, as well as different feeding rates.

The database presented in present study should be extended in order to cover other development stages for *A. baerii* a to elaborate a complete forecasting, based on long-time production cycle recorded data in order to contribute to the optimization of sturgeon’s aquaculture operational management.

The black-box soft sensor methodology developed in present study, based on MLR and GAM, should be applied in other technological scenarios as well, in order to develop the sensors applicability and to decrease the costs of real-time monitoring of water quality parameters within aquaponics systems.

## 3. Material and Methods

### 3.1. Experimental Design

**Figure 34:**
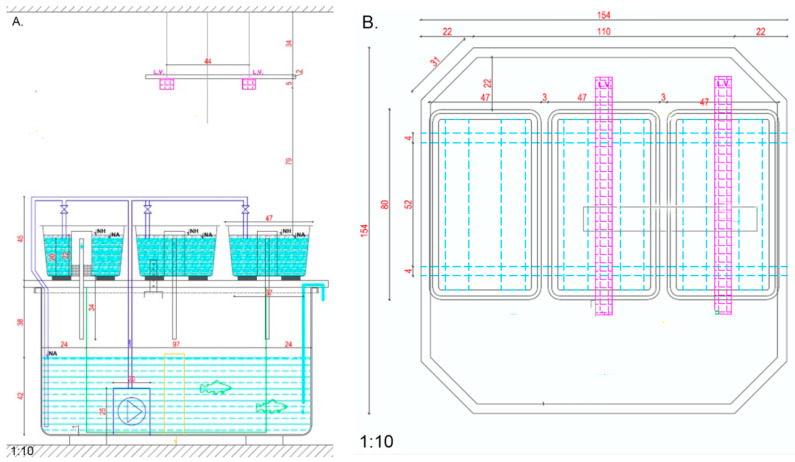
Aquaponics system layout as designed and constructed for the study ((**A**). cross section view, (**B**). top view).

Four RAS systems were simultaneously used for performing the experimental activities described in the present study, each with independent mechanical and biological filtration units and a water recirculation rate (Equation (11)) of 45%. The aquaponics modules were placed on the upper part of the fish-rearing units, as presented in Figure 34A,B.

A recirculating submerged pump with a screen case, placed in the interior of fish-rearing units, is used to assure a technological water recirculation loop cycle between RAS and the aquaponics modules, in continuous flow regime, assuring a hydraulic loading rate (Equation (12)) [88] of 6 m/day and a hydraulic retention time (Equation (13)) [88] of 0.69 h.

(11) $$r=\frac{Q\times 24}{V_{t}}\times 100$$

where *r* = water recirculation rate (%), *V_t_*—total hydraulic volume (m^3^).

(12) $$HLR=\frac{Q}{S}$$

where *HLR*—hydraulic loading rate [m/day], *Q*—recirculation flow for each aquaponic unit [m^3^/h], *S*—aquaponic unit surface area [m^2^].

(13) $$HRT=\frac{S\times h}{Q}$$

where *HRT*—hydraulic retention time [h], *h*—aquaponic unit water depth [m].

Each of the aquaponics culture units and fish tanks had an aeration tube placed within the GM, 15 cm above the bottom, in a round shape, to maintain dissolved oxygen (DO) concentrations to nearly full saturation and accentuate the mineralization process at the level of the GM. The technological water was not discharged during the study period. However, RO water was added in order to replenish evapotranspiration losses.

The photoperiod was 14 h during the first 7 days of the experimental period, followed by 12 h until the end of the production cycle, consisting of lighting using multi-spectrum lamps (21% blue, 38% green, 35% red, 6% far-red). Thus, the day period starts at 7:00 a.m. till 21:00 p.m. (in the first 7 days of the experiment), 19:00 p.m. (until the end of the production cycle). The air temperature recorded an average of 24 ± 1.94 °C during the day-time and 21 ± 1,.27 °C in the night-time, respectively, with an average relative humidity (RH) of 71.35 ± 6.01%.

The *Rapana venosa* shells GM was provided by a Romanian aquatic products processing Company named DeltaMar, from Cataloi city, Tulcea county. The rapana shells’ (R) by-products were manually sorted and the top of the shell was removed by breaking, in order to be able to eliminate all the organic debris using water jetting. The cleaned shells were then autoclaved at 100 °C for 1 h, to reduce the microbial load of these materials, as suggested in a previous research study [50], in the case of other by-products-based GM. The substrate was placed in the aquaponic units and covered by a thin layer of LECA (3 cm depth) in order to offer good growth stability to the basil seedlings. The conventional GM used as a reference in the present study consists of LECA.

The experimental design consists of 4 experimental variants in 3 replications, as follows: AH—high nutrients input and LECA GM, AR—high nutrients input and R GM, BH—low nutrients input and LECA GM, BR—low nutrients input and R GM. The nutrients input into the systems is exclusively dependent on the feeding rate since all variants started with the same water quality matrix: N-NH_4_ (0.01 mg/L), N-NO_2_ (0.03 mg/L), N-NO_3_ (2.21 mg/L), P-PO_4_ (0.02 mg/L), Ca (4.72 mg/L), Mg (1.64 mg/L), Fe (n.d.), K (0.879 mg/L), EC (337.81 μS/cm), pH (7.9). Thus, high nutrients experimental variants are associated with higher fish feed inputs (2484.61 g per variant, AH and AR, respectively), while lower nutrients inputs are corresponding to low fish feed inputs (1552.23 g per variant, BH and BR, respectively). Also, considering a previous study [89] that indicates Ca, Mg and K as main elements in the *Rappana* shell chemical composition, the R substrate was chemically analysed and the following results were obtained: Ca (27.13 mg/kg), Mg (447.10 mg/kg), K (117.22 mg/kg). However, in order to chemically interact with water, R GM must be exposed to a series of mechanical interactions that will result in shell erosion, fact that did not occurred in present study. Also, shells were exposed to the treatment protocol exposed above, fact that assured proper cleaning of R GM. The fish biomass used in the present experimental trial was provided by Silurus Market Company, Bucharest, Romania. Therefore, two groups of *Acipenser baerii* (A and B, respectively), in different development stages, with average individual biomass of 51 ± 5.3 g/ex for group A and 30.93 ± 3.72 g/ex for group B were divided into 2 experimental variants each, at a stocking density of 2.56 kg/m^3^ in the case of AH and AR and 1.55 kg/m^3^ in the case of BH and BR experimental variants. The fish biomass was fed, during the 43 days experimental trial, with Alltech Coppens^®^ feed, 56% protein and 15% fat by applying a daily feeding ratio of 1.5% biomass weight (BW). The *Ocimum basilicum* L. seedlings, with a quality certificate, were purchased from the Galati county local market, Romania. A culture density of 70 plants/m^2^ was applied in all the experimental variants.

### 3.2. The Evaluation of Both Basil (Ocimum basilicum L.) and Sturgeon (Acipenser baerii) Biomass Growth in Aquaponic Conditions Applied in Different Technological Scenarios

The total *Acipenser baerii* biomass was determined once every three days, during the entire experimental trial. Growth performance indicators were calculated as described in previous studies [90]. Both the shoot height and total leaf area of *Ocimum basilicum* L. were determined once every three days, as described in previous studies [49].

**Figure 35:**
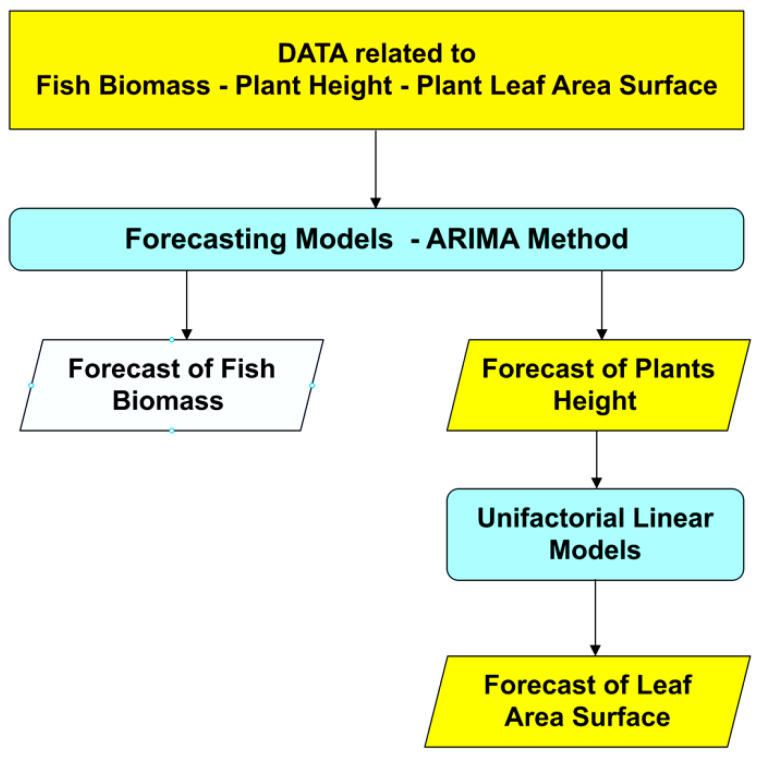
Analytical framework for data processing in order to elaborate fish and plants forecasting growth models.

The analytical framework for the forecasting of fish biomass, plants height and plants leaf area growth, used in present study, is revealed in Figure 35.

It is assumed that the evolution of various phenomena and scenarios is influenced by the generated output over a long period of time. Therefore, it can be assumed that a phenomenon that has reached a certain level of development has also created a base which will be used in the future for reaching, farther, at least at similar levels. Therefore, specific phenomena depend on their previous performances and are represented by auto-regression form. Driven by these particularities, the stochastic forecasting models were developed, which can be defined as follows:

(14) $$y_{t}=\bar{y}+a_{0}+a_{1}y_{t-1}+a_{2}y_{t-2}{}_{.}+\cdots +a_{p}y_{t-p}-b_{1}u_{t-1}-b_{2}u_{t-2}-\cdots -b_{q}u_{t-q}+u_{t}$$

where *y_t_* represents the stationary variable and *a*, *b*—the estimated parameters. 

The first part of Equation (14) represents the linear autoregressive model (*lag*), while the second part includes the error introduced through the residual values (*u_t_*_−*q*_), needed for correcting the forecast—it represents the mobile part of the model. The model represented by Equation (4) is an ARIMA type (*p*, *d*, *q*), where *p* represents the autoregressive part, *d* is the stationary order and *q* is the average mobile order.

Model identification—the first step in developing the model is the analysis of the time series. This implies the verification of series stationery. In case it is concluded that the series is not stationary, the next step will be to induce stationarity by transforming the data into differences. After the series becomes stationary, the next step is to check if correlations, autocorrelations and partial autocorrelations exist.

Parameters estimation—several forecasting models can be developed by using correlograms, autocorrelations (AC) and partial autocorrelations (PAC) coefficients which are aimed to estimate parameters using the least square method.

Choosing the model—considering the combinations between Auto-Regressive (AR) and Moving Average (MA) models, different forecasting ARIMA models can be developed, however, they must comply with certain conditions. In order to determine the model utility, the coefficient Akaike (Akaike information criteria—AIC) and the normality of the residual variable need to be checked. In the case of the AIC coefficient, the model for which the value of AIC is lowest shall be selected. Normality testing shall be determined by a graphic representation of the residual value, but also by using the coefficient Jarque-Berra.

The regression models are used to describe the existing relationship between one or more independent variables and a dependent variable. The regression analysis is the basis for many types of predictions, as well as for determining their effects on the target variable. In order to establish the relationship between variables, the use of a linear function is needed. Liniar regression is a model in which the relationship between inputs and outputs is a straight line and the points are situated near the line (Equation (15)).

(15) $$y_{t}=a+b_{1}x1_{t}+b_{2}x2_{t}+\cdots +b_{n}xn_{t}+u_{t}$$

where *y_t_* represents the target variable of the model, *xi_t_* are the independent variables of the model and *u_t_* represents the residuals or errors. The aim of regression models is to determine the best relationship that exists between the target variable and the independent one, which can be established by using the least square method.

In order to estimate the impact of plant height growth on leaf dimension, a linear factor model was used (Equation (16)), as it follows:

(16) $$leaf_{t}=a+b\;x\;height_{t}+u_{t}$$

where: *a*—represents the coefficient that indicates the influence of factors which were not included in the model, considered therefore as factors with constant influence (in present situation, the coefficient explain how much the leaf will grow if the plant will stagnate in height), *b*—the regression coefficient, indicates how much the leaf will increase or decrease in size based on an increase of the plant height by one unit, *leaf_t_* and *height_t_*—are model variables (one endogenous and the other exogenous) and *u_t_*—represents the model *noise*, namely the residual value which will be minimized by using the least squares method.

### 3.3. Multi Linear Regression (MLR) and Generalized Additive Models (GAM) for Developing Black-Box Soft Sensors for Water Quality Real-Time Monitoring

**Figure 36:**
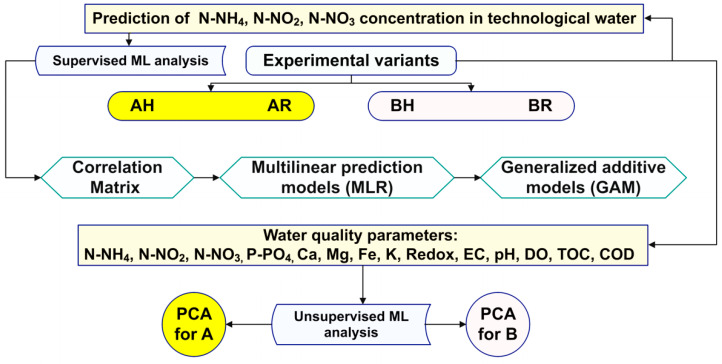
The analytical framework structure for developing water quality black-box sensors.

The analytical framework structure for developing water quality black-box sensors, based on unsupervised and supervised machine learning (ML), is presented in Figure 36.

The current research uses the *Multiple Linear Regression (MLR)* that allows to understand the existing relationship between a continuous dependent variable and two or more independent variables. The independent variables can be either continuous (like in the current research) or categorical that are supposed to be dummy coded before running any analysis. For the research models to be reliable and valid, the following essential requirements were verified: (a) the independent and dependent variables are linearly related, (b) there is no strong correlation between the independent variables, (c) Residuals have a constant variance, (d) Observations are independent of one another, (e) all variables follow multivariate normality. The purpose of multivariate multiple regression is to determine the line that best approximates the trend of the cloud-points of a distribution with several simultaneous variables. The regression equation (17) has the following form:

(17) $$Y=a+b_{1}X_{1}+b_{2}X_{2}+\cdots +b_{n}X_{n}$$

where: *Y* represents the dependent variable; *a* is the point of origin of the line; *b*_1_, *b*_2_…, *b_k_* are the estimators that will be determined for each individual predictor; *X*_1_, *X*_2_…, *X_k_* are the values of the *n* predictors.

After the identification of the trajectory that minimizes the estimation error, considering multiple correlations between the predictors (Pearson correlation), a coefficient of determination is calculated, which identifies the percentage of variation in the dependent variable determined by the simultaneous variation of the independent variables. An important aspect of the model is multicollinearity which represents the level of correlations between the independent variables. The determination of this hypothesis is carried out by identifying VIF coefficient (Variance Inflation Factor) whose value is targeted to be less than 5. Model validation is of particular importance and is done with the help of the multiple correlation coefficient and must have a maximum value for the sample for which the regression equation was calculated. If its value drops dramatically for another sample, then the determined regression equation does not show the utility that was estimated. A final aspect that was considered is the effect of extreme values (outliers) on the equation and therefore, before starting with the estimation of the equation, these limit values were first be identified.

The *generalized additive models (GAM)* were originally invented by Trevor Hastie and Robert Tibshirani [91] and provides a general framework for extending a standard linear model by allowing nonlinear functions of each variable while maintaining additivity. Such a model starts from the standard model by replacing each linear component *b_j_X_j_* with a non-linear function *f_j_*(*X_j_*) corresponding to feature *j* (Equation (18)), as follows:

(18) $$g^{-1}\left(E\left[Y\right]\right)=a+f_{1}\left(X_{1}\right)+f_{2}\left(X_{2}\right)+\cdots +f_{n}\left(X_{n}\right)+\varepsilon$$

where: *E*[*Y*] represents the arithmetic mean of the dependent variable *Y*; *g*^−1^ is the inverse of the function *g*, also called the link function; *a* is the point of origin of the trajectory; *f*_1_(*X*_1_), …, *f_n_*(*X_n_*) represents non-linear functions of the independent variables; *ε* is the error to be minimized. 

According to the described model, each function is calculated separately for each predictor, and then their contributions are added to the final result. The evaluation of *f_i_* functions is performed by interpolation, based on dispersion diagrams (scatterplot smoother), using cubic spline functions (Equation (19)), as follows:

(19) $$S\left(x\right)=a_{i}+b_{i}x+c_{i}x^{2}+d_{i}x^{3},\;pentru\;\forall x\in \left[x_{i-1},x_{i}\right];$$

where, S:[a,b]→ℝ; f:[a,b]→ℝ; (x_i_) = S(x_i_), $i=\bar{0,n}$; 

This framework is based on the following aspects: (a) the relationships between the dependent variable and the individual predictors follow smooth patterns that can be linear or nonlinear, (b) these smooth relationships can be simultaneously estimated and then predict the dependent variable by simply adding them up [92]. GAM represents an additive modeling technique able to capture the impact of the predictive variables through smooth functions which can be nonlinear, depending on the underlying patterns in the data. There are good reasons for using GAM in predictive problems [93]: (a) interpretability, (b) flexibility/automation, (c) regularization. Hence, if a model contains nonlinear effects (like in the current research), GAM provides a regularized and interpretable solution offering a good balance between the interpretable, yet biased, linear model, and the extremely flexible, “black box” learning algorithms. If a regression model is additive, the interpretation of the marginal impact of a single variable (the partial derivative) does not depend on the values of the other variables in the model. Thus, the output of the model provides insights related to the effects of the predictive variables. In addition, GAM models offer the possibility to control the smoothness of the predictor functions, thus avoiding predictor functions with too many inflexion points, by simply adjusting the level of smoothness [94]. It is possible to impose a prior belief that predictive relationships are inherently smooth in nature, even though the dataset at hand may suggest a noisy relationship.

Principal component analysis (PCA)—The PAC performed stages are as follows: Vector projections–Eigen values and Eigen vectors–Lagrange Multipliers–Derivative’s of a matrix–Covariance matrix. The Vector projections stage targets to determine the line F1 that passes through the origin and best fits the point cloud. Each length of the projection of the line on F1 is the scalar product of the point X with the unit vector (*U*_1_) (Equation (20)). To adjust the cloud of points, the method of least squares was used and the sum of the squares of the projections was maximized.

(20) $$\frac{1}{n}\sum_{i=1}^{n}{\left(U_{1}^{T}X_{i}-U_{1}^{T}\bar{X}\right)}^{2}=\frac{1}{n}\sum_{i=1}^{n}{\left[U_{1}^{T}\left(X_{i}-\bar{X}\right)\right]}^{2}=U_{1}^{T}SU_{1}\;\max U_{1}^{T}SU_{1}\;\mathrm{cu}\;\mathrm{condi}ț\mathrm{ia}\;U_{1}^{T}U_{1}=1$$

where: *U*_1_ is the unit vector; *U*_1_*^T^* is the unit vector transpose; *X_i_* the vectors of the independent variables; $\bar{X}$ is the arithmetic mean of the vectors; *S* is the covariance matrix.

### 3.4. Water Quality Analysis

The Libelium^®^ Smart Water Sensor Platform Adds Ion Monitoring (Zaragoza, Spain) was used in order to assure real-time monitoring of nitrate, nitrite, ammonia, magnesium (Mg), calcium (Ca), pH, conductivity (EC), dissolved oxygen (DO) and temperature and transmit the data for visualization and cloud storage, via Waspmode^®^ (Zaragoza, Spain), to Grafana^®^ platform—developed by Grafana Labs Company (New York, NY, USA). The orthophosphate (PO_4_), TOC and COD were measured by using Merck Spectroquant^®^ (Darmstadt, Germany) test kits. The ORD was measured by using a Hach HQ1110 portable ORP meter (Düsseldorf, Germany). Iron (Fe) and potassium (K) were measured using the flame atomic absorption spectrometry (FAAS) technique. In this sense, water samples were filtered (0.45 µm filter size), mineralised with nitric acid (HNO_3_ 65% Suprapur) and analysed by using the high-resolution continuum source atomic absorption spectrometer (HR-CS-AAS) ContrAA 700 by Analytik Jena (Jena, Germany). The N-NH_4_, N-NO_2_ and N-NO_3_ reduction rates were calculated as described in previous research papers [50,95].

### 3.5. Plant Quality Analysis

The concentrations of Ca, Mg and K in plant leaves were determined by using the FAAS technique. After harvesting, each plant was sampled in triplicate (n = 3) and approximately 1 g of biomass was extracted. The cations were extracted after the digestion procedure, in nitric acid (HNO_3_ 65% Suprapur) and hydrogen peroxide (H_2_O_2_ 30% Emsure). The digestion was performed in a 5-step programme specific to vegetable leaves by using the micro-wave assisted pressure digestion system Top Wave by Analytik Jena. Further on, the digested samples were diluted with deionized water and the target cations were determined by using the HR-CS-AAS ContrAA 700 by Analytik Jena (Jena, Germany). The nitrate levels, in both basil roots and leaves, were determined using the Griess method (STAS 9065:2002). Sweet basil biomass, representing aerial parts and roots belonging to all experimental variants were dried in dark at room temperature and further extracted with methanol by 30 min ultrasound-assisted extraction. After filtration, extracts were made up to 10 mL with methanol and kept at −20 °C until studied. Folin-Ciocalteu’s phenol reagent, gallic acid, 2,2-diphenyl-1-picrylhydrazyl radical (DPPH) and rutin were purchased from Sigma-Aldrich^®^ (Steinheim, Germany). Sodium nitrite was obtained from Riedel-de Haën^®^ (Seelze, Germany). All other chemicals and reagents were of analytical grade. Total phenolic content was quantified using the Folin-Ciocalteu assay as previously described [96,97] with minor changes. In brief, 0.2 mL of each extract were mixed with 3 mL of distilled water and 0.2 mL of Folin-Ciocalteu reagent. A volume of 0.6 mL of 20% sodium carbonate was added after 5 min followed by vigorous shaking and 2 h incubation at room temperature in dark. Finally, the absorbance was read at 765 nm (Specord 210 Plus spectrophotometer, Analytik Jena, Jena, Thuringia, Germany). Total phenolic content was expressed as g of gallic acid equivalents per 100 g of biomass. The experiments were performed in triplicate and the results were expressed as mean value ± standard deviation.

Flavonoids were quantified spectrophotometrically by aluminium chloride assay as previously described [96,97] with slight modifications. A volume of 0.5 mL of each extract was mixed with distilled water (1 mL) and 5% sodium nitrite (0.075 mL) followed by subsequent addition of 0.15 mL of 10% aluminium chloride (after 6 min) and 0.5 mL of 1 M sodium hydroxide (after other 5 min). The absorbance of the reaction mixture was determined at 765 nm (Specord 210 Plus spectrophotometer, Analytik Jena, Jena, Thuringia, Germany). Flavonoid content was expressed as g of rutin equivalents per 100 g of biomass. The experiments were performed in triplicate and the results were expressed as mean value ± standard deviation.

DPPH scavenging activity of basil extracts was assessed according to a described methodology [97,98] with minor changes. An aliquot of 0.5 mL of each extract was mixed with 1.5 mL of DPPH in methanol (A517 nm = 1.00 ± 0.05). After 5 min incubation, the absorbance was read at 517 nm (Specord 210 Plus spectrophotometer, Analytik Jena, Jena, Thuringia, Germany). DPPH radical scavenging activity (%) was calculated as follows: 100 × (Ainitial − Afinal)/Ainitial, where Ainitial and Afinal are the absorbances before and after 5 min incubation with extracts. All experiments were carried out in triplicate; the results were expressed as mean value ± standard deviation.

## 4. Conclusions

The present research manages to elaborate an analytical framework, based on a holistic approach, in order to optimize both the environmental and economical sustainability of aquaponic sturgeon (*Acipenser baerii*)—basil (*Ocimum basilicum* L.) integrated recirculating systems. Thus, it can be concluded that the use of innovative R GM assures better performances in terms of basil growth, compared to conventional H GM, situation emphasized by the superior values of basil individual biomass, recorded at the end of the production cycle and associated to AR and BR experimental variants, compared to AH and BH.

However, in terms of both phenolic and flavonoids content in basil leaves and roots biomasses, the variants based on H GM records superior values, compare to those based on R GM with one exception manifested at A technological scenario, where AR recorded better values than AH if analyzing the root biomass. The DPPH from basil leaves emphasizes higher values associated to R GM, fact valid in both technological scenarios (A and B). The concentration of K, Mg, Ca and NO_3_ reveal the highest values at R GM variants, compared to H GM and at A technological scenario, compared to B scenario, respecrively.

The forecasting models for *A. baerii* growth indicates ARIMA (1, 2, 1), (1, 2, 2), (2, 2, 1), (2, 2, 2) as suitable to be applied for A technological scenario, while ARIMA (1, 2, 1), (1, 2, 2), (2, 2, 1), (2, 2, 2), (1, 2, 3), (2, 2, 3), (3, 2, 1), (3, 2, 2) and (3, 2, 3) are recommended to be used for B technological scenario. The basil height and leaves surface dynamics is forecasted accurately with ARIMA (1, 1, 1,), (1, 1, 2), (2, 1, 1) and (2, 1, 2) in the case of AH and AR experimental variants, while ARIMA (1, 3, 1), (1, 3, 2), (2, 3, 1), (2, 3, 2), (3, 3, 1), (3, 3, 2) is used at BH and ARIMA (1, 2, 1), (1, 2, 2), (2, 2, 1), (2, 2, 2) at BR.

In terms of N-NH_4_ and N-NO_3_ reduction rate, the H GM variants assure better performance compared to R GM, in each of the technological scenarios.

The MLR and GAM prediction models reveal the highest metrics accuracy when predicting N-NO_3_ concentration in technological water, emphasizing the opportunity of developing future studies in order to define a N-NO_3_ soft sensor, based on data recorded during longer-time experimental periods. The PCA analysis reveals a high correlation between pH and DO in the case AR, AH and BH, situation which can be considered when future studies that implies research for developing technological water quality soft sensors for aquaponics systems.

## 5. Patents

Patent request recorded at OSIM, no. A/00671/24.10.2022, entitled “Aquaponic system with growth media for sustainable growth of *Acipenser baerii* and *Ocimum basilicum* L.”.

## Figures and Tables

**Table 1:** The *p*-values obtained after ADF test, by using square root differences for *A. baerii* biomass time series data.

Number of Differences	ADF Test (*p*-Value) for Series A	ADF Test (*p*-Value) for Series B
0	0.9825	1.0000
1	0.5752	0.6230
2	0.0407	0.0008

**Table 2:** The Akaike coefficient of tested ARIMA models, based on *A. baerii* time series data.

Model	Akaike Coefficient for Series A Model	Akaike Coefficient for Series B Model
ARIMA(1,2,1)	0.143	0.642
ARIMA(1,2,2)	0.325	−3.019
ARIMA(1,2,3)	-	−0.947
ARIMA(2,2,1)	0.325	0.719
ARIMA(2,2,2)	−4.010	0.363
ARIMA(2,2,3)	-	0.264
ARIMA(3,2,1)	-	−0.849
ARIMA(3,2,2)	-	−1.305
ARIMA(3,2,3)	-	−1.391

**Table 3:** The *p*-values obtained after ADF test, by using square root differences, for basil heigh time series data.

Number of Differences	ADF Test (*p*-Value) for AH Series	ADF Test (*p*-Value) for AR Series	ADF Test (*p*-Value) for BH Series	ADF Test (*p*-Value) for BR Series
0	0.6099	0.1373	0.3670	0.7138
1	0.0360	0.0119	0.1006	0.2367
2	-	-	0.4085	0.0020
3	-	-	0.0000	-

**Table 4:** The Akaike coefficient for the tested ARIMA models, based on basil heigh time series data.

Model	Akaike Coefficient for Series AH Model	Akaike Coefficient for Series AR Model	Akaike Coefficient for Series BH Model	Akaike Coefficient for Series BR Model
ARIMA (1, 1, 1)	−0.53	−6.25	-	-
ARIMA (1, 1, 2)	−5.38	−5.91	-	-
ARIMA (1, 2, 1)	-	-	-	−5.19
ARIMA (1, 2, 2)	-	-	-	−5.83
ARIMA (1, 3, 1)	-	-	−5.13	-
ARIMA (1, 3, 2)	-	-	−5.10	-
ARIMA (2, 1, 1)	−5.29	−4.96	-	-
ARIMA (2, 1, 2)	−5.13	−7.06	-	-
ARIMA (2, 2, 1)	-	-	-	−6.63
ARIMA (2, 2, 2)	-	-	-	−8.07
ARIMA (2, 3, 1)	-	-	−4.83	-
ARIMA (2, 3, 2)	-	-	−6.77	-
ARIMA (3, 3, 1)	-	-	−4.98	-
ARIMA (3, 3, 2)	-	-	−5.32	-

**Table 5:** The average concentration of main water quality parameters recorded at both inlet and outlet of aquaponics modules corresponding to each experimental variant.

Water Quality Parameter	Concentrations Recorded in Sampling Points of Each Experimental Variant *							
	AH		AR		BH		BR	
	Inlet	Outlet	Inlet	Outlet	Inlet	Outlet	Inlet	Outlet
N-NH_4_ (mg/L)	0.07 ± 0.05 ^a^	0.06 ± 0.04 ^b^	0.12 ± 0.08 ^c^	0.09 ± 0.06 ^d^	0.07 ± 0.08 ^a^	0.06 ± 0.07 ^b^	0.06 ± 0.04 ^b^	0.05 ± 0.03 ^e^
N-NO_2_ (mg/L)	0.10 ± 0.05 ^a^	0.08 ± 0.04 ^b^	0.12 ± 0.07 ^c^	0.10 ± 0.06 ^a^	0.06 ± 0.02 ^d^	0.05 ± 0.02 ^e^	0.13 ± 0.09 ^f^	0.10 ± 0.07 ^a^
N-NO_3_ (mg/L)	21.84 ± 9.33 ^a^	21.19 ± 9.05 ^b^	28.69 ± 12.22 ^c^	28.11 ± 11.98 ^d^	7.75 ± 2.14 ^e^	6.86 ± 1.87 ^f^	10.51 ± 3.79 ^g^	10.09 ± 3.64 ^h^
P-PO_4_ (mg/L)	3.75 ± 1.47 ^a^	3.32 ± 1.07 ^b^	4.97 ± 1.68 ^c^	4.27 ± 1.10 ^d^	2.47 ± 0.98 ^e^	1.96 ± 0.86 ^f^	2.85 ± 0.95 ^g^	2.45 ± 1.02 ^e^
Ca (mg/L)	38.41 ± 1.81 ^a^	36.43 ± 1.70 ^b^	40.83 ± 1.94 ^c^	39.19 ± 2.52 ^d^	21.15 ± 1.23 ^e^	19.93 ± 1.73 ^f^	21.80 ± 1.28 ^g^	20.92 ± 1.31 ^h^
Mg (mg/L)	16.80 ± 2.22 ^a^	15.99 ± 2.30 ^b^	17.65 ± 2.28 ^c^	16.90 ± 2.08 ^a^	11.28 ± 1.29 ^d^	10.85 ± 1.13 ^e^	11.73 ± 1.39 ^e^	11.39 ± 1.19 ^f^
Fe (mg/L)	0.14 ± 0.08 ^a^	0.03 ± 0.06 ^b^	0.19 ± 0.07 ^c^	0.05 ± 0.07 ^d^	0.09 ± 0.03 ^e^	0.01 ± 0.04 ^f^	0.11 ± 0.05 ^g^	0.02 ± 0.05 ^h^
Redox (mV)	80.13 ± 17.39 ^a^	92.45 ± 15.76 ^b^	78.26 ± 18.33 ^c^	88.34 ± 19.72 ^d^	77.12 ± 14.56 ^c^	84.70 ± 16.62 ^e^	79.60 ± 16.04 ^c^	86.90 ± 12.75 ^e^
K (mg/L)	5.57 ± 0.55 ^a^	5.07 ± 0.31 ^b^	8.04 ± 0.34 ^c^	7.85 ± 0.42 ^d^	5.41 ± 0.52 ^e^	5.21 ± 0.43 ^f^	5.51 ± 0.33 ^g^	5.27 ± 0.39 ^h^
EC (μs/cm)	1235.17 ± 168.94 ^a^	1168.47 ± 154.18 ^b^	1262.79 ± 168.25 ^a^	1209.95 ± 170.36 ^c^	1181.28 ± 168.96 ^b^	1110.45 ± 172.98 ^d^	1227.99 ± 170.95 ^a^	1166.62 ± 166.90 ^b^
pH (upH)	6.51 ± 0.19 ^a^	6.35 ± 0.19 ^b^	6.70 ± 0.20 ^c^	6.41 ± 0.14 ^d^	6.88 ± 0.22 ^e^	6.67 ± 0.18 ^c^	6.57 ± 0.22 ^a^	6.40 ± 0.14 ^d^
DO (mg/L)	7.67 ± 0.67 ^a^	7.53 ± 0.67 ^b^	8.04 ± 0.64 ^c^	7.98 ± 0.64 ^d^	7.72 ± 0.67 ^e^	7.68 ± 0.67 ^a^	8.09 ± 0.64 ^f^	8.07 ± 0.63 ^c^
TOC (mg/L)	143.58 ± 72.11 ^a^	135.17 ± 88.23 ^b^	112.67 ± 57.23 ^c^	108.23 ± 67.20 ^d^	93.58 ± 76.30 ^e^	84.76 ± 54.89 ^f^	79.23 ± 67.12 ^g^	70.18 ± 58.34 ^h^
COD (mg/L)	113.50 ± 72.13 ^a^	104.70 ± 59.34 ^b^	108.60 ± 52.34 ^c^	99.34 ± 63.11 ^d^	40.40 ± 33.14 ^e^	38.60 ± 21.11 ^f^	43.30 ± 36.23 ^g^	39.1 ± 29.23 ^h^

* Different letters on the same line reveal significantly statistical differences (*p* < 0.05).

**Table 6:** The MLR models for predicting nitrogen compounds concentration in technological water, for each of the experimental variants.

Crt. No.	Experimental Variant	MLR Prediction Model	Rsq.	Adj. Rsq.	Root Mean Square Error (RMSE)
1.	AH	N-NH_4_ = −0.142 + 0.005 Ca + 0.028 DO + 0.004 Mg − 0.012 N-NO_2_ − 0.015 pH	0.644	0.579	0.025
2.	AH	N-NO_2_ = −0.735 + 0.004 Ca + 0.105 DO − 0.019 N-NH_4_ + 0.005 N-NO_3_ − 0.038 pH	0.442	0.339	0.032
3.	AH	N-NO_3_ = 82.818 +1.492 Ca − 12.16 DO − 0.009 EC + 0.257 Mg − 2.387 N-NH_4_ + 20.980 N-NO_2_ − 2.865 pH	0.947	0.938	2.058
4.	AR	N-NH_4_ = −0.108 + 0.004 Ca + 0.028 DO + 0.005 Mg + 0.148 N-NO_2_ + 0.003 N-NO_3_ + 0.007 pH	0.472	0.374	0.052
5.	AR	N-NO_2_ = −0.571 + 0.003 Ca + 0.067 DO + 0.152 N-NH_4_ + 0.002 N-NO_3_ − 0.008 pH	0.268	0.133	0.052
6.	AR	N-NO_3_ = 67.478 + 0.442 Ca − 8.526 DO + 0.028 EC + 0.842 Mg + 31.738 N-NH_4_ + 20.077 N-NO_2_ − 6.671 pH	0.809	0.775	5.166
7.	BH	N-NH_4_ = 0.006 − 0.017 Ca + 0.077 DO + 0.014 Mg + 0.385 N-NO_2_ − 0.078 pH	0.488	0.394	0.051
8.	BH	N-NO_2_ = −0.185 + 0.008 Ca + 0.039 DO + 0.004 Mg + 0.034 N-NH_4_ + 0.009 N-NO_3_ − 0.039 pH	0.505	0.414	0.015
9.	BH	N-NO_3_ = 19.645 − 0.016 Ca − 3.644 DO − 0.231 Mg − 0.081 N-NH_4_ + 24.379 N-NO_2_ + 2.681 pH	0.846	0.819	0.789
10.	BR	N-NH_4_ = 0.077 + 0.007 Ca + 0.006 DO + 0.004 Mg + 0.112 N-NO_2_ − 0.015 pH	0.543	0.459	0.025
11.	BR	N-NO_2_ = −0.840 + 0.007 Ca + 0.091 DO + 0.003 Mg + 0.645 N-NH_4_ + 0.012 N-NO_3_ − 0.028 pH	0.389	0.277	0.059
12.	BR	N-NO_3_ = 47.337 + 0.137 Ca − 2.895 DO + 0.007 EC − 0.259 Mg + 4.036 N-NH_4_ + 8.915 N-NO_2_ − 3.612 pH	0.804	0.768	1.612

## Data Availability

The data which are not contained within the research paper in raw format can be available upon request from the corresponding author (I.A.S.—ira.simionov@gmail.com).
